# Cooling intact and demembranated trabeculae from rat heart releases myosin motors from their inhibited conformation

**DOI:** 10.1085/jgp.202113029

**Published:** 2022-01-28

**Authors:** Jesus G. Ovejero, Luca Fusi, So-Jin Park-Holohan, Andrea Ghisleni, Theyencheri Narayanan, Malcolm Irving, Elisabetta Brunello

**Affiliations:** 1 Randall Centre for Cell and Molecular Biophysics, King’s College London, London, UK; 2 Centre for Human and Applied Physiological Sciences, King’s College London, London, UK; 3 European Synchrotron Radiation Facility, Grenoble, France

## Abstract

Myosin filament–based regulation supplements actin filament–based regulation to control the strength and speed of contraction in heart muscle. In diastole, myosin motors form a folded helical array that inhibits actin interaction; during contraction, they are released from that array. A similar structural transition has been observed in mammalian skeletal muscle, in which cooling below physiological temperature has been shown to reproduce some of the structural features of the activation of myosin filaments during active contraction. Here, we used small-angle x-ray diffraction to characterize the structural changes in the myosin filaments associated with cooling of resting and relaxed trabeculae from the right ventricle of rat hearts from 39°C to 7°C. In intact quiescent trabeculae, cooling disrupted the folded helical conformation of the myosin motors and induced extension of the filament backbone, as observed in the transition from diastole to peak systolic force at 27°C. Demembranation of trabeculae in relaxing conditions induced expansion of the filament lattice, but the structure of the myosin filaments was mostly preserved at 39°C. Cooling of relaxed demembranated trabeculae induced changes in motor conformation and filament structure similar to those observed in intact quiescent trabeculae. Osmotic compression of the filament lattice to restore its spacing to that of intact trabeculae at 39°C stabilized the helical folded state against disruption by cooling. The myosin filament structure and motor conformation of intact trabeculae at 39°C were largely preserved in demembranated trabeculae at 27°C or above in the presence of Dextran, allowing the physiological mechanisms of myosin filament–based regulation to be studied in those conditions.

## Teaser

Ovejero et al. show that cooling resting or relaxed rat heart muscle releases myosin motors from the helical folded conformation that inhibits actin interaction in physiological conditions.

## Introduction

The contraction of striated muscle is generated by relative sliding of actin-containing thin filaments and myosin-containing thick filaments driven by myosin motors and is initiated by an intracellular calcium transient elicited by an action potential (Bers, 2002). Calcium binding to troponin switches on the thin filaments by triggering the movement of tropomyosin around the filament that unmasks the actin sites to which the myosin motors can bind and power contraction (Gordon et al., 2000). Recent EM studies have elucidated the molecular basis of these regulatory structural changes in the cardiac thin filament triggered by calcium binding to troponin at saturating (Yamada et al., 2020) and submaximal calcium concentrations (Risi et al., 2021). However, the myosin motors are not immediately available for interaction with actin, as they are folded into helical tracks on the surface of the thick filament in an asymmetric conformation called the “interacting heads motif” (Zoghbi et al, 2008; AL-Khayat et al, 2013), which was first identified in smooth muscle myosin (Wendt et al., 2001) and in isolated thick filaments from skeletal muscle of the tarantula (Woodhead et al., 2005). This folded conformation is assumed to inhibit actomyosin interaction and myosin adenosine triphosphatase, locking the myosin motors in an energy-saving state that has been identified with the “super-relaxed state” observed biochemically (Stewart et al., 2010; Hooijman et al., 2011), although other factors may also modulate that state (Chu et al., 2021). Recent studies on cardiac muscle using x-ray diffraction (Reconditi et al., 2017; Brunello et al., 2020) and fluorescent probes on myosin (Park-Holohan et al., 2021) showed that in the resting phase of the cardiac cycle between beats, called “diastole,” the myosin motors are folded onto the thick filament surface in a conformation consistent with the interacting heads motif. These findings led to the idea that the actomyosin interaction in cardiac muscle is controlled by a dual-filament mechanism in which calcium binding to the thin filament regulates the availability of the actin-binding sites for myosin (Gordon et al., 2000; Lehman, 2016; Tobacman, 2021), whereas the myosin filament controls the number of myosin motors that are available for interaction with actin (Irving, 2017).

Thick filament–based regulation is thought to be disrupted by mutations in thick filament proteins associated with heart disease (Alamo et al., 2017; Trivedi et al., 2018; Nag and Trivedi, 2021), so it represents a potential target for new treatments (Green et al., 2016; Toepfer et al., 2020). However, the mechanisms that control the release of the myosin motors from the folded state during muscle contraction are still not understood. Recent studies suggested that the cardiac thick filament can be directly switched on by the filament stress (Reconditi et al., 2017; Brunello et al., 2020; Park-Holohan et al., 2021), in a mechano-sensing mechanism similar to that described in skeletal muscle (Linari et al., 2015; Fusi et al., 2016). In cardiac muscle of the rat, mechano-sensing seems to require submaximal calcium activation of the thin filaments, suggesting that a calcium-dependent interfilament signaling pathway controls the activation of the thick filament (Park-Holohan et al., 2021), although apparently conflicting results have been reported for porcine heart (Ma et al., 2021b).

In resting or relaxed mammalian skeletal muscle, myosin motors can be released from the helical folded conformation by cooling below physiological temperature (Wray, 1987; Malinchik et al., 1997; Xu et al., 1997; Xu et al., 1999; Caremani et al., 2019; Caremani et al., 2021), and this protocol has been widely used to characterize this structural transition in the absence of calcium and force. The decreased helical order on cooling is thought to be mediated by the transition from the ADP.Pi or switch-2 closed state of the myosin motor to the ATP or switch-2 open state (Xu et al., 2003; Ma et al., 2021a), but is also accompanied by an increase in the axial periodicity of the myosin motors and the filament backbone. Fluorescence polarization studies using probes on the myosin regulatory light chain in relaxed skeletal muscle fibers showed that the light chain domain or lever arm of the myosin motors becomes more perpendicular to the filament axis on cooling, consistent with loss of the folded conformation (Fusi et al., 2015).

Disruption of the helical order of the thick filament has been also reported in relaxed demembranated cardiac trabeculae cooled from 25°C to 5°C (Martyn et al., 2004; Xu et al., 2006). More recently, a fluorescence polarization study using probes on the regulatory light chain of myosin in relaxed cardiac trabeculae showed that the fraction of myosin motors in the folded conformation is maximized at near physiological temperature and lattice spacing and that cooling favors myosin light chain domain orientations more perpendicular to the filament axis (Park-Holohan et al., 2021).

Here, we used x-ray diffraction to characterize the structural changes in the thick filament associated with cooling intact quiescent trabeculae from 39°C to 7°C. We show that the myosin motors leave the helical folded state on cooling, and the periodicity of the thick filament backbone increases. Because cooling intact trabeculae alters their intracellular ionic composition, we also characterized the effect of cooling on thick filament structure in relaxed demembranated cardiac trabeculae, in which the ionic composition of the solution bathing the myofilaments is controlled. Addition of the osmotic agent Dextran T500 (3% wt/vol) to the relaxing solution restored the filament lattice spacing to that in intact muscle cells at 39°C and stabilized the folded helical conformation of the myosin motors against the effects of cooling.

## Materials and methods

### Preparation of cardiac trabeculae and experimental protocol

The cardiac trabeculae were prepared as previously described (Brunello et al., 2020). Briefly, Wistar Han rats (*Rattus norvegicus*, male, 6–8 wk old) were supplied by Charles River Laboratories and hosted at the animal house of the Bio-Medical Facility of the European Synchrotron Radiation Facility (ESRF). On the day of the experiment, the rats were euthanized by cervical dislocation after sedation with isoflurane in compliance with the UK Home Office Schedule 1 and European Union regulation (directive 2010/63), followed by a confirmation method. The heart was rapidly excised and cannulated via the ascending aorta and retrogradely perfused with a modified Krebs-Henseleit buffer (119 mM NaCl, 5 mM KCl, 0.5 mM CaCl_2_, 1.2 mM NaH_2_PO_4_, 1.2 mM MgSO_4_, 25 mM NaHCO_3_, 10 mM glucose, and 25 mM 2,3-Butanedione oxime [BDM]) equilibrated with carbogen (95% O_2_, 5% CO_2_). The pH of the Krebs solution decreased from ∼7.5 at 36°C to ∼7.3 at 7°C. Single unbranched trabeculae were dissected from the right ventricle under a stereomicroscope and were selected to be used as intact or demembranated samples.

**Figure S1.**
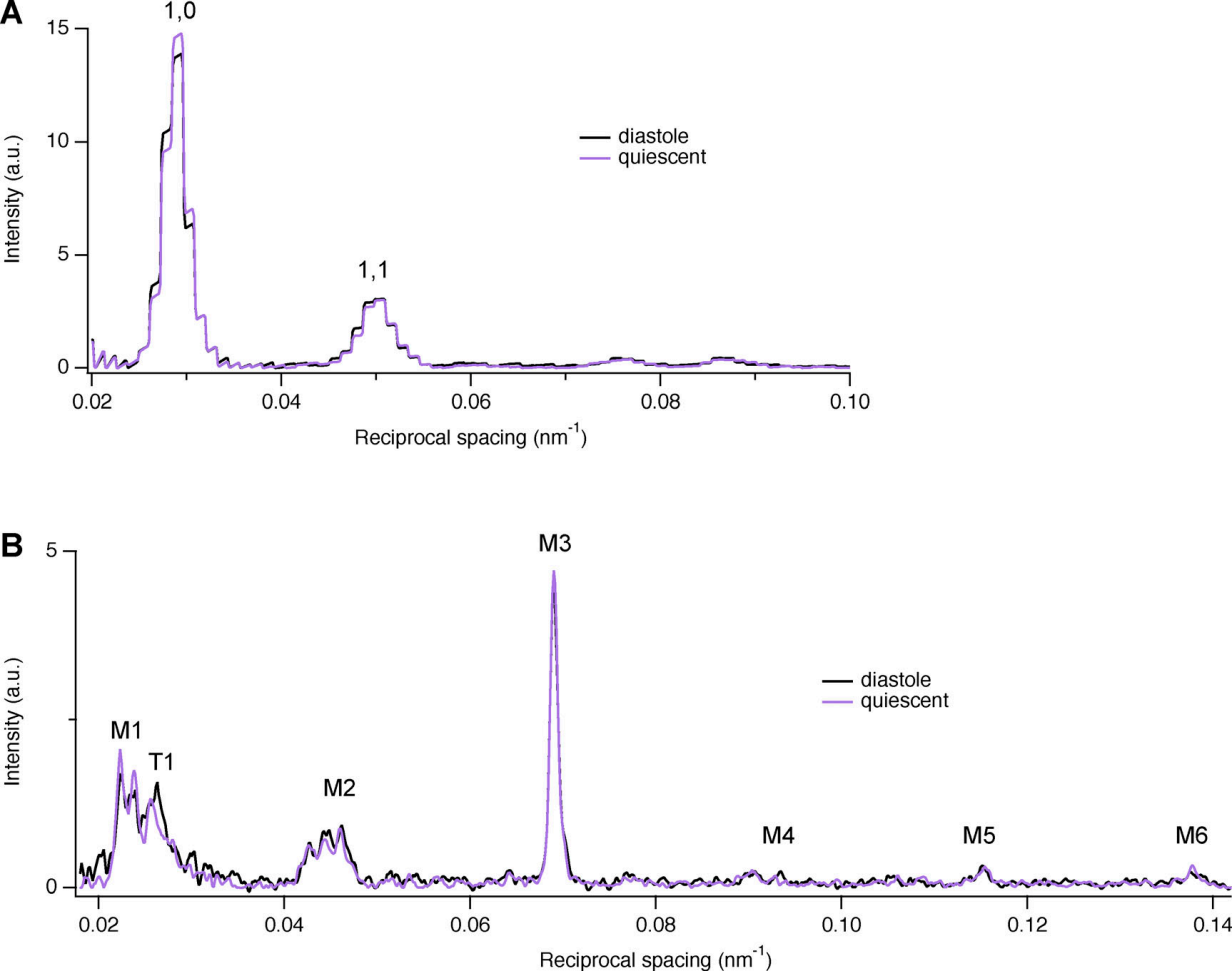
**Equatorial and meridional intensity distributions** **from the same trabecula either quiescent or in the diastolic phase between beats.** **(A and B)** Equatorial (A) and meridional (B) intensity distributions (black; stimulation frequency, 1 Hz; purple, quiescent). Temperature, 27°C. The steplike appearance of the equatorial reflections is due to the horizontal 8× binning of the detector pixels before readout as explained in Materials and methods. Total exposure time, 40 ms. a.u., arbitrary unit.

**Figure S2.**
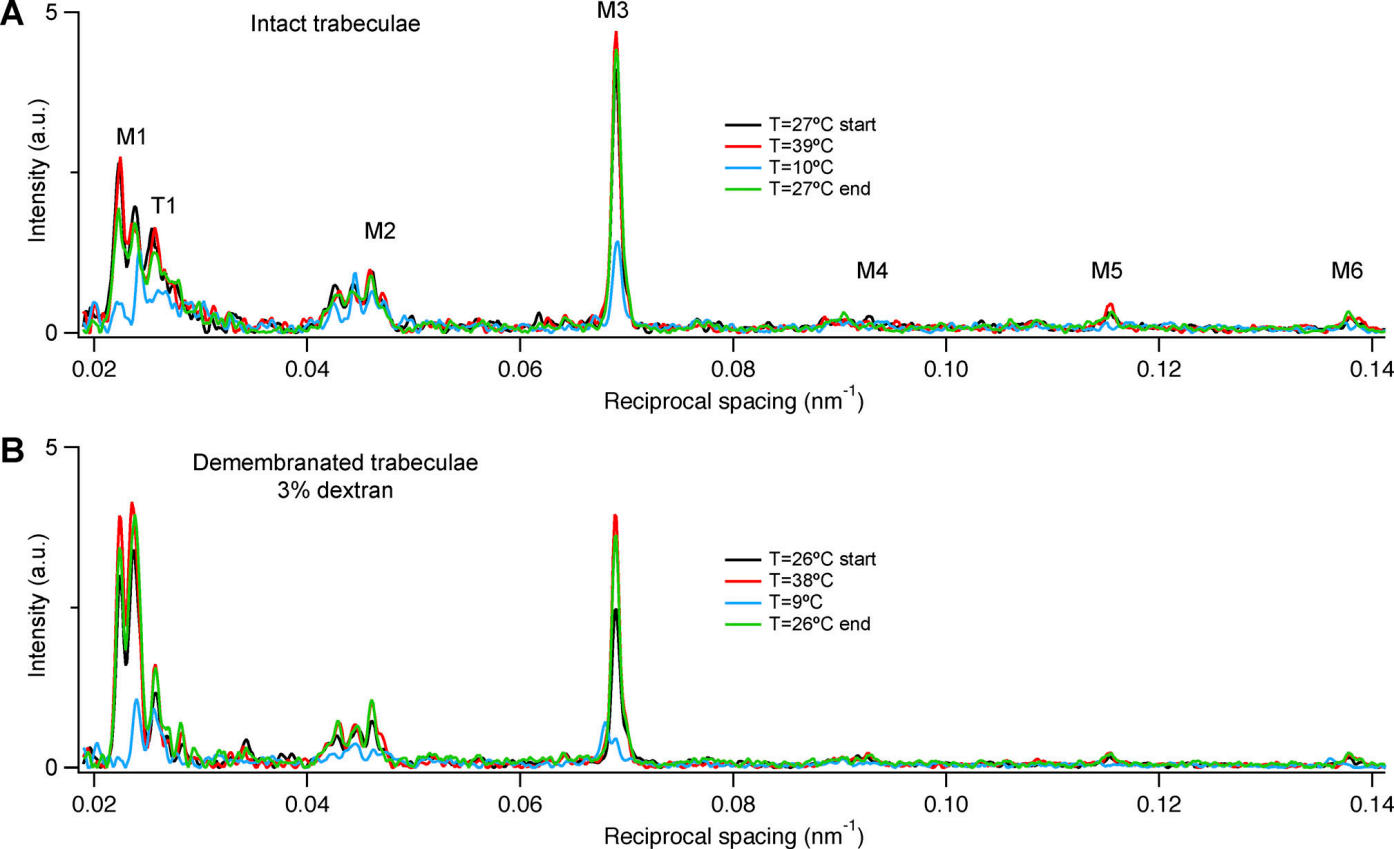
**Reversibility of the effect of temperature on the structure of the thick filament.** **(A and B)** Meridional intensity distributions at 27°C before (black trace) and after (green trace) high (39°C, red trace) and low temperature (10°C, blue trace) in one intact (A) and one demembranated trabecula in the presence of Dextran (B). Total exposure time, 40 ms. a.u., arbitrary unit.

For experiments on intact trabeculae, “scorpion-like” clips made of stainless-steel wire were mounted on the valve and ventricular wall ends of each trabecula. Trabecular length and cross-sectional area were measured after stretching to just above slack length. They were then mounted in the experimental plexiglass trough filled with the same buffer between the levers of a strain gauge force transducer and a motor (322C; Aurora Scientific Inc.) at slack length. The trabecula was then perfused with a buffer containing 1.4 mM CaCl_2_ without BDM at 27.6 ± 0.1°C (mean ± SD, *n* = 5 trabeculae). Two mica windows carrying platinum-stimulating electrodes were positioned as close as possible to the trabecula to minimize the x-ray path in solution, and the trabeculae were electrically paced at 1 Hz for at least 30 min before the start of the experiment to check the viability of the preparation. Continuous perfusion keeps the level of oxygen constant at the sample position. The temperature inside the trough was controlled by adjusting the temperature of the perfusate via a counter-current system and continuously monitored with a temperature probe. The speed of temperature change was very slow when warming/cooling due to the overall inertia of the system (10–20 min); therefore, cooling did not induce spontaneous contractures (Milani-Nejad et al., 2014). However, the trabeculae did not respond to electrical stimulation below 17°C, as previously reported (de Tombe and ter Keurs, 1990); therefore, the full temperature range was investigated in quiescent trabeculae. At 27°C, the x-ray pattern in the quiescent state was not significantly different from that in the diastolic phase between beats (Fig. S1; see also Reconditi et al., 2017). In some trabeculae, the lower temperatures induced disruption of thick filament structure; therefore, data for the lowest experimental temperature were always recorded before the last control at 27°C; an example of the reversibility of the temperature effect on the meridional intensity distributions in one intact trabecula is shown in Fig. S2 A. Sarcomere length (SL) was measured at multiple points along the trabecula by ultra-small–angle x-ray diffraction at 31-m sample-to-detector distance, and the average SL was set to 2.05 ± 0.06 µm. Average trabecular cross-sectional area was 50,300 ± 23,400 µm^2^ (mean ± SD), and length was 2.64 ± 0.57 mm.

**Figure S3.**
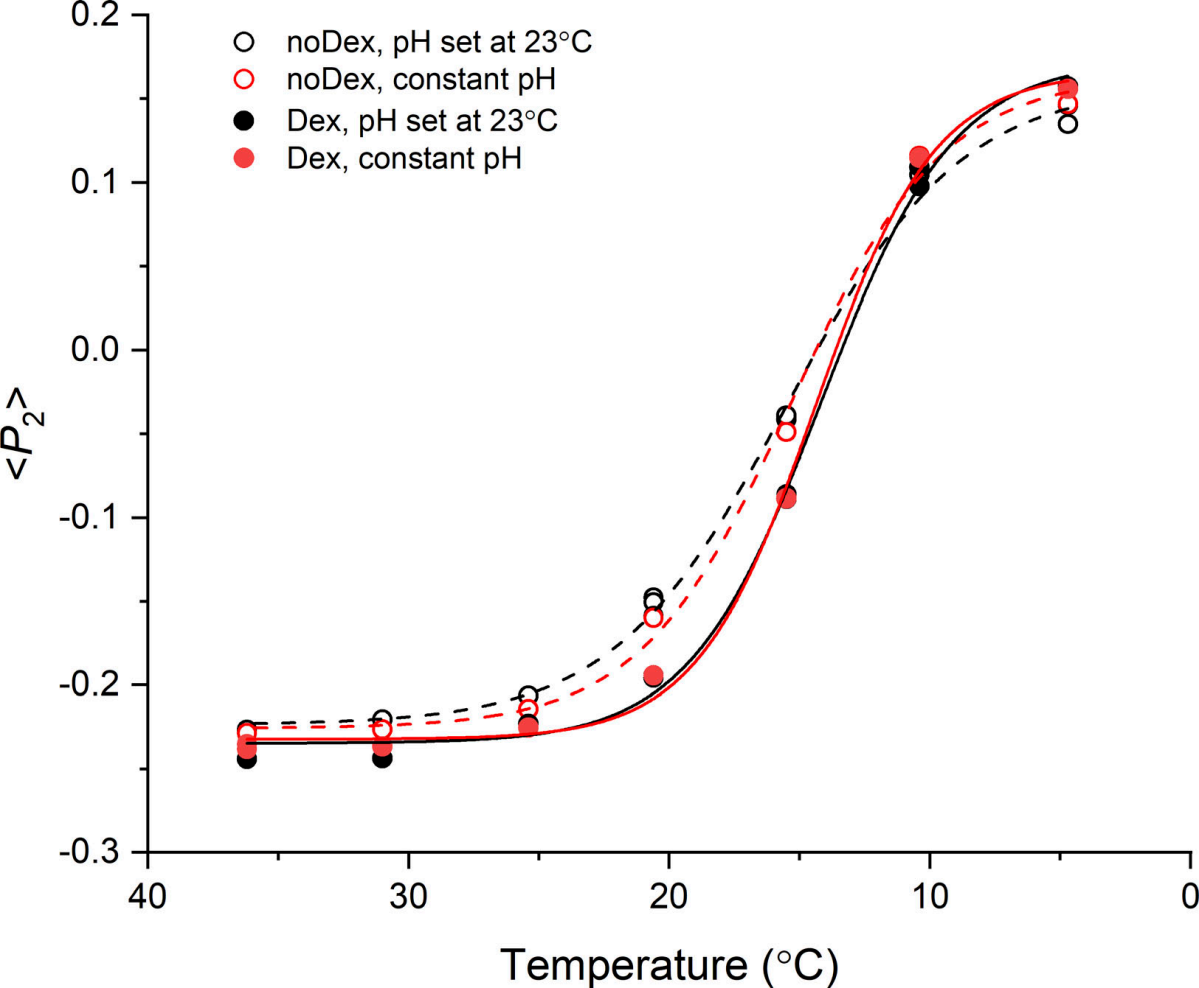
**Temperature and pH dependence of the orientation of the myosin regulatory light chain in relaxed trabeculae.** Orientation of a bifunctional rhodamine probe cross-linking helices B and C in the N-lobe of the regulatory light chain of myosin (BC probe; Park-Holohan et al., 2021), expressed by the order parameter <*P*_2_>, in a cardiac trabecula incubated in relaxing solution with pH set at 7.1 at 23°C (black) and at constant pH = 7.1 (red), in the absence (empty symbols and dashed lines; noDex) or in the presence (filled symbols and continuous lines; Dex) of 3% Dextran (wt/vol). Lines are Boltzmann fits to the data.

For experiments on demembranated trabeculae, isolated trabeculae were demembranated for 20 min on ice in relaxing solution in the presence of BDM (25 mM) and Triton X-100 1% (vol/vol), then stored at −20°C in storage solution (6 mM Imidazole, 70 mM KPr, 8 mM MgAc_2_, 5 mM EGTA, 7 mM Na_2_ATP, 1 mM NaN_3_, and 50% glycerol) for up to 24 h. Aluminum T-clips were attached to the ends of the trabeculae, and they were washed in relaxing solution before mounting in a temperature-controlled multidrop apparatus (Fusi et al., 2014) in relaxing solution at SL 2.12 ± 0.02 µm (mean ± SD, *n* = 4 trabeculae) between the levers of a strain gauge force transducer and the Aurora motor. Relaxing solution contained 25 mM Imidazole, 45 mM KPr, 6.89 mM MgAc_2_, 10 mM EGTA, 5.56 mM Na_2_ATP, 20 mM Na_2_-creatine phosphate, (pCa = −log [Ca^2+^] = 9), free [Mg^2+^] = 1.0 mM, ionic strength = 180 mM, and pH 7.1 at 23°C. In the same trabeculae, the osmotic agent Dextran T500 (3% wt/vol) was added to the relaxing solution to reduce the interfilament spacing to a value similar to that of intact trabeculae at body temperature. Just before the experiment Protease inhibitor cocktail P8340 (Sigma) and 2 mM dithiothreitol was added to all the solutions. The temperature of the multidrop trough was controlled by a Peltier system and recorded with a temperature probe. Due to the very small volume of each drop and the high thermal conductance of the aluminum base, the speed of temperature change was fast (<1 min). The reversibility of the effect of cooling on the meridional x-ray intensity distribution in demembranated trabeculae is shown in Fig. S2 B. The pH of the relaxing solution was 6.85 at 36°C and 7.45 at 9°C. The effect of this pH change on myosin motor conformation was assessed using a bifunctional rhodamine probe in the N-lobe of the regulatory light chain, which is sensitive to the loss of the helically ordered state on cooling (Park-Holohan et al., 2021). The probe order parameter <*P*_2_> has a temperature dependence similar to that of the x-ray signatures of the helical folded state of the myosin motors reported below, and its value at each temperature was not significantly affected when the pH of the relaxing solution was set to 7.1 at each temperature (Fig. S3). Thus, the conformation of the myosin motors is independent of the pH changes in the present x-ray measurements with demembranated trabeculae, as found previously in skeletal muscle (Fusi et al., 2015). However, the pH changes induced by cooling may alter the charge on the filaments and the filament lattice spacing (Millman, 1998). Average trabecular cross-sectional area was 37,800 ± 10,900 µm^2^ (mean ± SD), and length was 1.63 ± 0.51 mm.

### X-ray data collection

The x-ray experiments were performed at beamline ID02 of the ESRF, which provided up to 2 × 10^13^ photons s^−1^ at 0.1-nm wavelength in a beam of size 300 µm (horizontal, full width at half-maximum) and 50 µm (vertical) at the detector (Narayanan et al., 2018). The trabeculae were mounted vertically at the beamline to increase the spatial resolution of the meridional reflections (parallel to the trabecular axis): the trough for intact trabeculae was sealed to prevent solution leakage before going vertical, while the multidrop trough for the demembranated trabeculae was mounted vertical and the drops were held between the coverslips by capillarity. X-ray patterns from demembranated trabeculae were collected with the trabecula in air to avoid x-ray absorption by the glass coverslips. The beam was attenuated to 3% (25-µm Fe attenuator) for trabecular alignment. To minimize radiation damage, x-ray exposure was limited to the data collection period using a fast electromagnetic shutter, and the trabecula was moved vertically by 100–200 µm between exposures. Maximum full-beam exposure time at each position was either 10 or 20 ms. Data were collected at the seven different temperature values (7/9, 11/13, 16/18, 22, 28/26, 32/31, and 39/38°C, intact/demembranated trabeculae, respectively) in each trabecula and at three different points along each sample at each temperature. X-ray diffraction patterns were recorded using the high-spatial resolution FReLoN CCD detector (Narayanan et al., 2018; ESRF), with active area 50 mm × 50 mm, 2,048 × 2,048 pixels, pixel size 24 µm × 24 µm, positioned either 1.6 m from the trabecula to record x-ray signals up to the sixth order of the myosin meridional reflections or at 31 m to measure SL. At 1.6-m sample-to-detector distance, x-ray patterns were binned by 8 in the horizontal direction before readout to increase the signal-to-noise ratio. Data from four or five trabeculae were added for Fig. S5, Fig. S6, and Fig. S7 to further increase the signal-to-noise ratio of the M1/T1 and M2 reflection clusters.

### X-ray data analysis

Small angle x-ray diffraction data were analyzed using the SAXS package (P. Boesecke, ESRF), Fit2D (A. Hammersley, ESRF), and IgorPro (WaveMetrix, Inc.). 2-D patterns were centered and aligned using the equatorial 1,0 reflections, then mirrored horizontally and vertically. For each trabecula, data obtained at the same temperature were added to increase signal-to-noise for the weakest reflections.

Equatorial intensity distributions were obtained by integrating from 0.0036 nm^−1^ on either side of the equatorial axis (perpendicular to the trabecular axis), and the intensities and spacings of the 1,0 and 1,1 reflections were determined by fitting two Gaussian peaks in the region 0.019–0.071 nm^−1^ with the constraint *d*_11_ = *d*_10_/$\sqrt{3}.$ Equatorial data with adequate signal-to-noise could be obtained from single trabeculae.

Meridional intensity distributions were obtained by integrating from 0.0045 nm^−1^ on either side of the meridional axis (parallel to the trabecular axis). Background intensity distributions were fitted using a convex hull algorithm and subtracted; the small residual background was removed using the intensity distribution from a nearby region of the pattern containing no reflections. The interference components of the meridional reflections were determined by fitting multiple Gaussian peaks to the meridional intensity distribution from the following axial regions: M1-T1, 0.034–0.017 nm^−1^, four Gaussian peaks, the two lower angles with the same width for M1, the two higher angles with another width for T1; M2 cluster, 0.054–0.036 nm^−1^, four Gaussian peaks, the two lower angles with the same width for M2L, the two higher angles with another width for M2H (Caremani et al., 2021); M3, 0.060–0.076 nm^−1^, two or three Gaussian peaks with the same width; M6, 0.129–0.144 nm^−1^, one or two Gaussian peaks with the same width. The interference components of the M3 reflection in Fig. 4, A and B were obtained with a multiple Gaussian fit in which the starred peak was fitted with a different axial width from that of the three peaks fitted to the main M3 reflection. The cross-meridional width of the M3 reflection was determined from the radial distribution of its intensity in the axial region defined above using a double-Gaussian function centered on the meridian, with the wider component considered to be background. The combined instrumental point spread function was negligible compared with the radial width of the M3 reflection. The cross-meridional width of the M6 reflection was determined from the average radial profile obtained at each temperature by adding the 1-D profiles from four or five trabeculae after normalization for the mass in the beam, estimated using *I*_10_ at 27°C. The total intensity of each meridional reflection was calculated as the sum of the component peaks, and the spacing of the reflection as the weighted average of the axial spacing of the component peaks. The spacing was calibrated using the spacing of the M3 reflection (*S*_M3_) = 14.479 nm measured in intact trabeculae in diastole and in demembranated trabeculae at low calcium concentration (pCa 9) at 27°C (Brunello et al., 2020). Intensities of the M3 and M6 reflections were corrected for changes in their respective cross-meridional width.

The first myosin layer line (ML1) was integrated radially in the region between 0.063 and 0.023 nm^−1^ from the meridional axis. These 1-D profiles were then integrated axially in the region 0.017–0.024 nm^−1^ to exclude the contribution of the first actin layer line.

**Figure 1.**
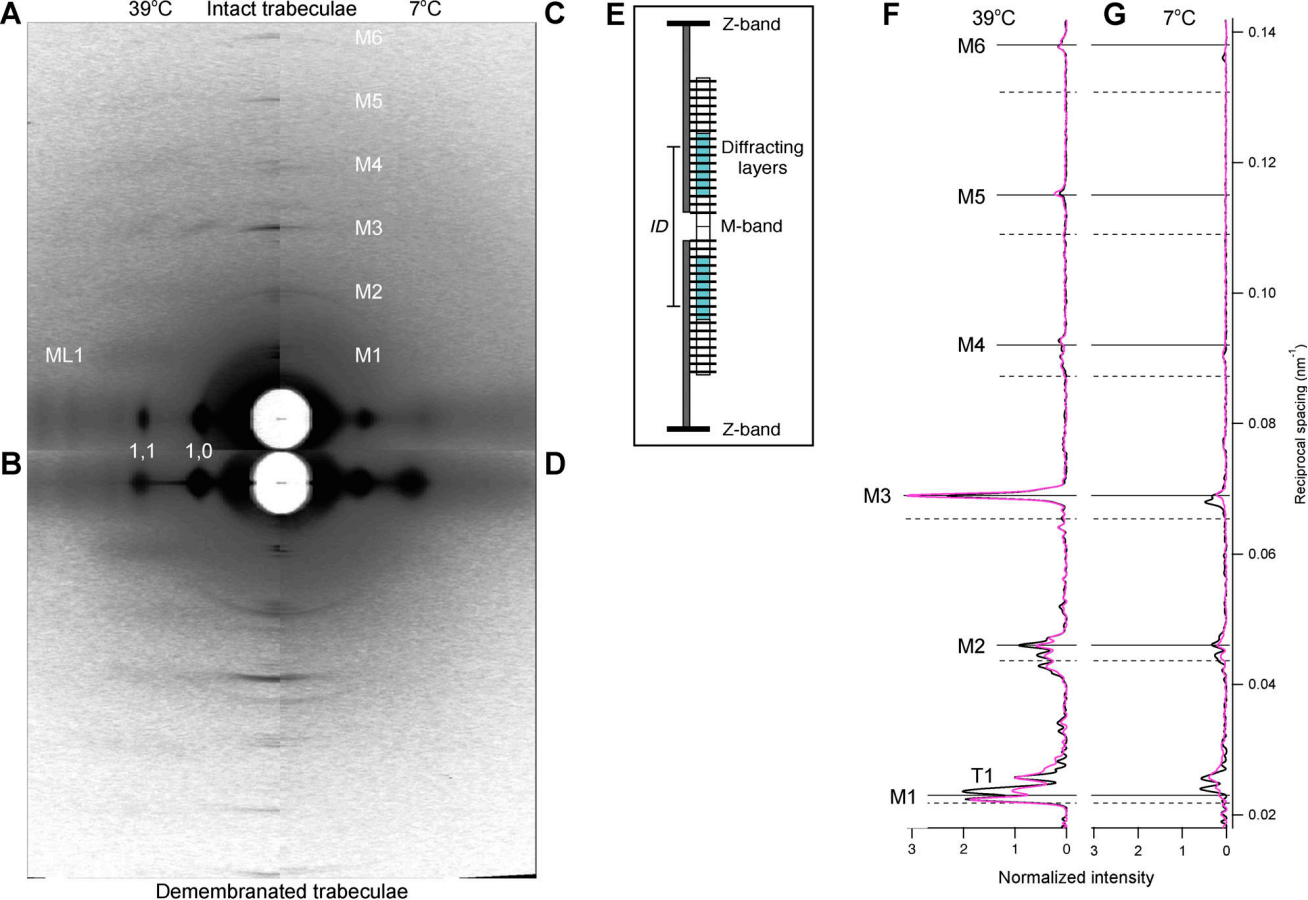
**X-ray diffraction patterns in isolated trabeculae. (A–D)** 2-D x-ray diffraction pattern in intact quiescent trabeculae (A and C) and in relaxed demembranated trabeculae in the presence of 3% Dextran (B and D). Temperature, 38–39°C (A and B) and 7–9°C (C and D). FReLoN detector; sample-to-detector distance, 1.6 m. Data added from *n* = 4 intact or demembranated trabeculae, respectively. Total exposure time, 160 ms. **(E)** Schematic of the cardiac sarcomere, delimited by Z-bands (black), contains overlapping actin (dark gray) and myosin filaments (white). MyBP-C–containing C-zone, cyan; layers of myosin motors (horizontal lines on myosin filament). M-band, myosin filament midpoint. **(F and G)** Meridional intensity distributions at 38–39°C (F) and 7–9°C (G) from the intact and demembranated trabeculae in A and C (magenta line), and B and D (black line), respectively. Data normalized by the main peak of the T1 reflection at 38–39°C for both intact and demembranated trabeculae. Continuous and dashed horizontal lines indicate orders of the shorter (higher-angle, H) and longer (lower-angle, L) myosin periodicities at high temperature (Table S1), respectively.

**Figure 2.**
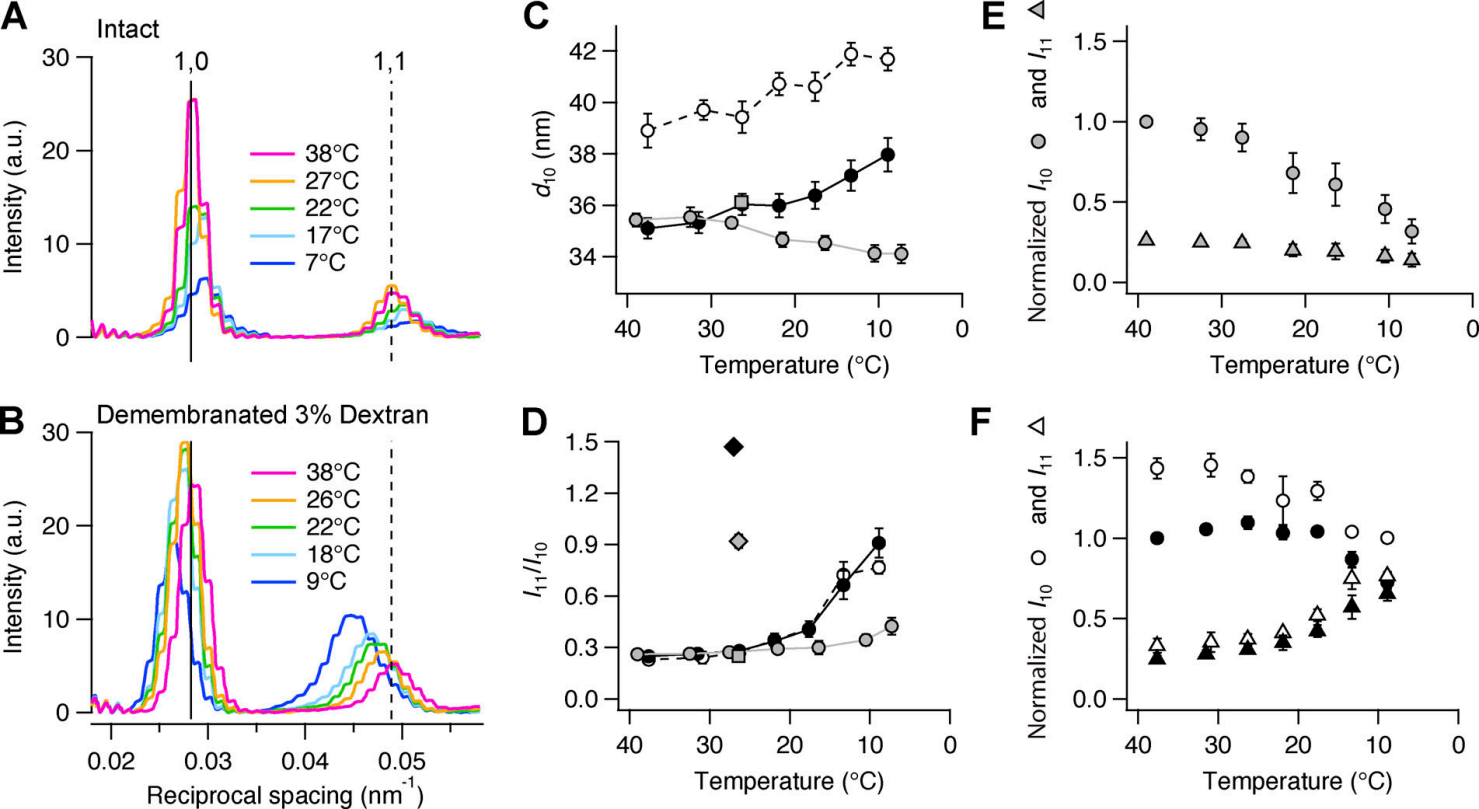
**Effect of cooling on the equatorial reflections. (A)** Equatorial intensity distributions (perpendicular to the trabecular axis) from one intact trabecula at five temperatures. Magenta, 39°C; orange, 27°C; green, 22°C; cyan, 17°C; blue, 7°C. **(B)** Corresponding profiles in one demembranated trabecula in the presence of 3% Dextran. Total exposure time, 40 ms. Vertical continuous and dashed lines, position of 1,0 and 1,1 reflections in intact trabecula at 38°C, respectively. **(C and D)** Spacing of 1,0 reflection (*d*_10_) and ratio of equatorial intensities (*I*_11_/*I*_10_). Gray circles and line, intact trabeculae; empty circles and dashed black line, demembranated trabeculae in the absence of Dextran; black circles and continuous line, demembranated trabeculae in the presence of 3% Dextran. Mean ± SE; *n* = 5 intact trabeculae and *n* = 4 demembranated trabeculae. Gray square in C and D, diastolic value in intact trabeculae paced at 1 Hz (T = 26.4°C; data from Brunello et al., 2020); gray diamond in D, peak force value in intact trabeculae (T = 26.4°C; data from Brunello et al., 2020); black diamond in D, maximal calcium activation ([Ca^2+^] = 20 µM; 3% Dextran; T = 27°C; from Brunello et al., 2020). **(E and F)** Intensity of 1,0 (*I*_10_, circles) and 1,1 (*I*_11_, triangles) reflections from intact (E; gray symbols) and demembranated trabeculae (F; black symbols, 3% Dextran; white symbols, no Dextran). Mean ± SE; *n* = 5 intact trabeculae and *n* = 4 demembranated trabeculae. Data in E normalized by *I*_10_ at 39°C; data in F normalized by *I*_10_ at 38°C in the presence of Dextran. a.u., arbitrary unit.

**Figure 3.**
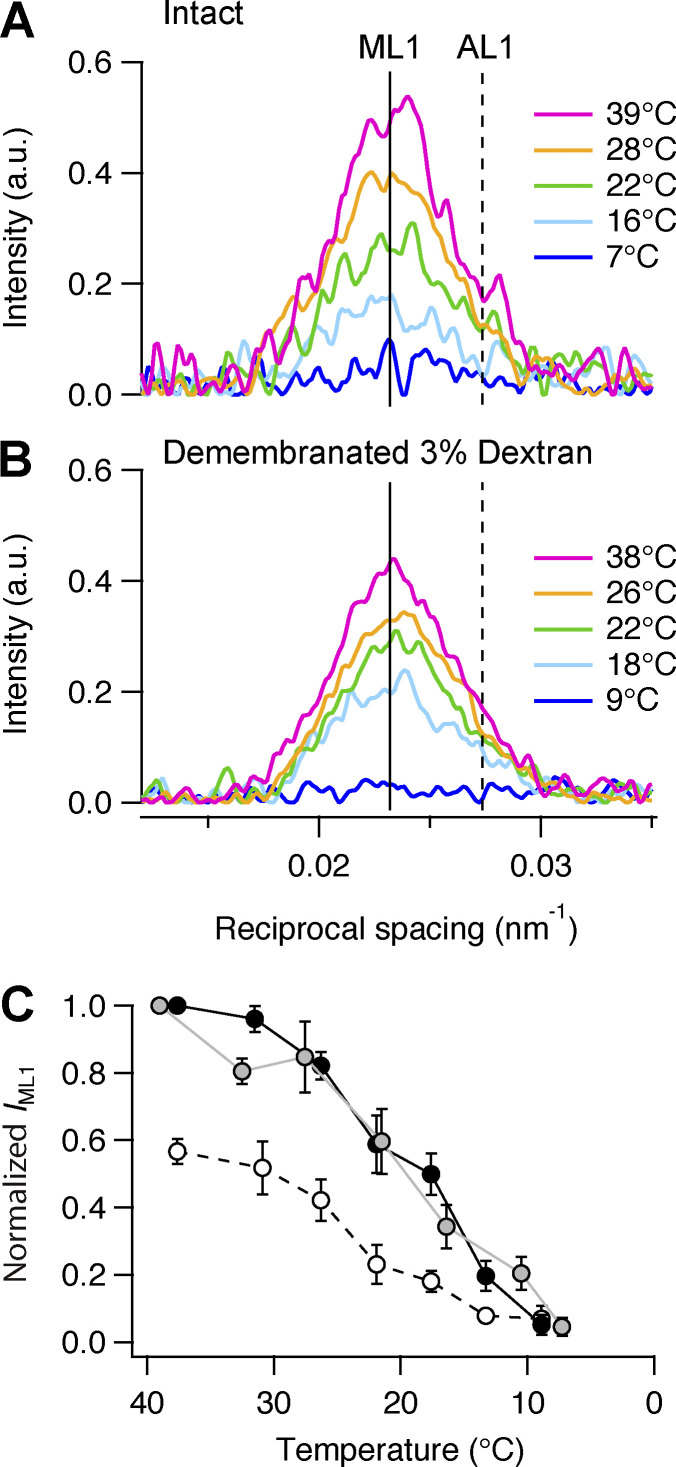
**Effect of cooling on the first myosin layer line (ML1). (A)** Axial profiles (parallel to the meridional axis) of the mixed myosin (ML1) and actin (AL1) layer line reflection in intact trabeculae at five relevant temperatures. Magenta, 39°C; orange, 28°C; green, 22°C; cyan, 16°C; blue, 7°C. Data added from *n* = 5 intact trabeculae. **(B)** Corresponding profiles in demembranated trabeculae in the presence of Dextran. Data added from *n* = 4 demembranated trabeculae. Vertical continuous and dashed line, ML1 and AL1 spacings, respectively, as estimated in Brunello et al. (2020). **(C)** Intensity of ML1 reflection (*I*_ML1_). Gray circles and line, intact trabeculae; empty circles and dashed black line, demembranated trabeculae in the absence of Dextran; black circles and continuous line, demembranated trabeculae in the presence of 3% Dextran. Mean ± SE; *n* = 5 intact trabeculae and *n* = 4 demembranated trabeculae. Data for intact trabeculae are normalized by the *I*_ML1_ value at 39°C. Data for demembranated trabeculae are normalized by the *I*_ML1_ value at 38°C in the presence of Dextran. a.u., arbitrary unit.

**Figure 4.**
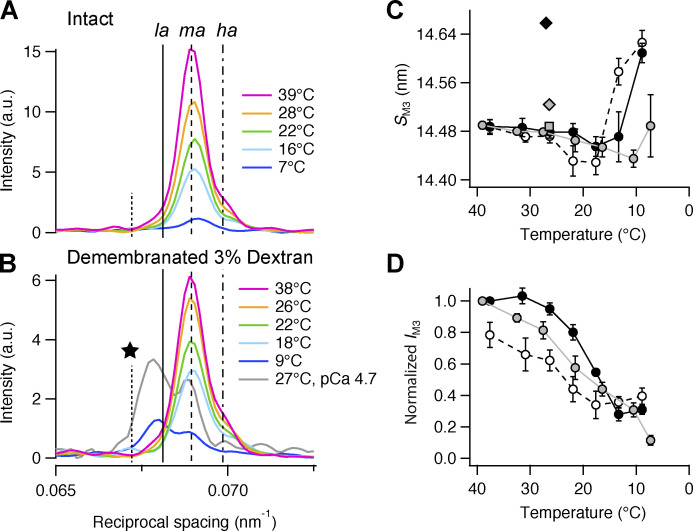
**Effect of cooling on the M3 reflection. (A)** Axial profiles of the M3 reflection (parallel to the trabecular axis) in intact trabeculae at five relevant temperatures. **(B)** Corresponding profiles in demembranated trabeculae in the presence of Dextran. Color code and number of trabeculae as in Fig. 3, A and B; gray trace in B is the axial profile of M3 reflection during maximal calcium activation (pCa 4.7; T = 27°C) scaled with respect to its axial profile at pCa 9.0 at 27°C; data from Brunello et al., 2020. Vertical lines mark the position of the three component peaks of the M3 reflection in intact trabeculae at 39°C (continuous line, *la*; dashed line, *ma*; dashed-dotted line, *ha*) and the additional lower angle peak (star) that was only visible at selected temperatures below 20°C and is not considered to be part of the M3 reflection. **(C and D)** M3 spacing (*S*_M3_; C) and intensity (*I*_M3_; D). Data in D are corrected by the radial width and normalized by the value at 39°C and 38°C in intact trabeculae and in demembranated trabeculae in the presence of Dextran, respectively. Gray circles and line, intact trabeculae; empty circles and dashed black line, demembranated trabeculae in the absence of Dextran; black circles and continuous line, demembranated trabeculae in the presence of 3% Dextran. Mean ± SE; *n* = 5 intact trabeculae and *n* = 4 demembranated trabeculae. Gray square and diamond in C, diastolic and systolic peak force values, respectively, in intact trabeculae paced at 1 Hz (T = 26.4°C; data from Brunello et al., 2020); black diamond in C, maximal calcium activation ([Ca^2+^] = 20 µM; 3% Dextran; T = 27°C; data from Brunello et al., 2020). a.u., arbitrary unit.

**Figure 5.**
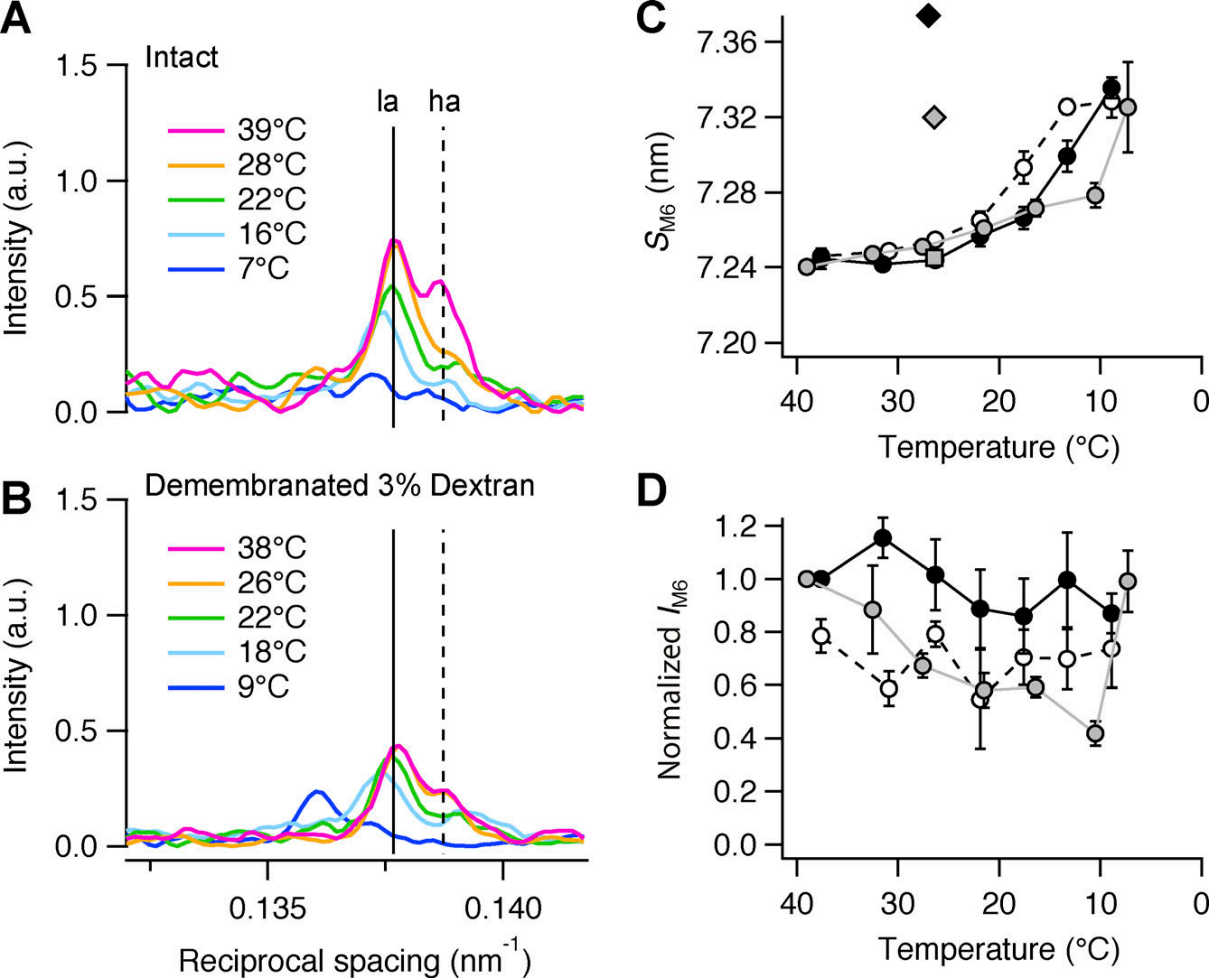
**Effect of cooling on the M6 reflection. (A)** Axial profiles of the M6 reflection in intact trabeculae at five relevant temperatures. **(B)** Corresponding profiles in demembranated trabeculae in the presence of Dextran. Color code and number of trabeculae as in Fig. 3, A and B. Vertical dashed lines mark the position of the two component peaks of the reflection in intact trabeculae at 39°C. **(C and D)** M6 spacing (*S*_M6_; C) and intensity (*I*_M6_; D). Data in D are corrected by the radial width and normalized by the value at 38°C in intact trabeculae and in demembranated trabeculae in the presence of Dextran. Gray circles and line, intact trabeculae; empty circles and dashed black line, demembranated trabeculae in the absence of Dextran; black circles and continuous line, demembranated trabeculae in the presence of 3% Dextran. Mean ± SE; *n* = 5 intact trabeculae and *n* = 4 demembranated trabeculae. Gray square and diamond in C, diastolic and systolic peak force values, respectively, in intact trabeculae paced at 1 Hz (T = 26.4°C; data from Brunello et al., 2020); black diamond in C, maximal calcium activation ([Ca^2+^] = 20 µM; 3% Dextran; T = 27°C; data from Brunello et al., 2020). a.u., arbitrary unit.

1-D profiles shown in Fig. 1, F and G; Fig. 2, A and B; Fig. 3, A and B; Fig. 4, A and B; Fig. 5, A and B; Fig. S5, A and B; and Fig. S7, A and B were added from four or five trabeculae after normalization for the mass in the beam, estimated using *I*_10_ at 27°C.

Force, stimulus, fiber length change, and x-ray acquisition timing were collected and analyzed using custom-made software written in LabVIEW (National Instruments).

### Calculation of the interference distance from the myosin-based meridional reflections

Myosin filaments from cardiac muscle are centrosymmetric about their midpoint at the M-band of the sarcomere (see Fig. 1 E) and contain two arrays of myosin motors with an axial periodicity *d* ∼14.5 nm. Each of the arrays contains 49 layers of three dimeric myosin molecules, or six motor domains. The thick filament was represented as a 1-D lattice of point diffractors with mirror symmetry about the filament midpoint, with the first lattice point in each half-filament at the half-bare zone (*hbz*), followed by the other 48 layers. *hbz* is an estimate of the average center of mass of the myosin motors; therefore, it can be used to determine changes in motor conformation; however it holds an uncertainty of ± 7.2 * *i* nm, where *i* is an integer (Reconditi et al., 2011; Hill et al., 2021). The axial profile of the M3 reflection was calculated as (Reconditi, 2006) *I*(*R*) = [sin(*N*π*Rd*)/sin(π*Rd*)]^2^cos^2^(π*RD*), where *R* is the reciprocal space coordinate, *d* is the axial spacing of the myosin layers, *N* is the number of contributing myosin layers in a contiguous array in each half-filament, from a proximal layer *n*_p_ to a distal layer *n*_d_, while motors outside that array are isotropic and make no contribution to the reflection; *D* is the interference distance (*ID*) calculated as *D* = 2 * *hbz *+ (*n*_p_ + *n*_d_ − 2) * *d* (Hill et al., 2021). The calculated *I*(*R*) was convoluted with a Gaussian function with sigma ∼50 µm, representing the combined point-spread function of the x-ray beam and detector, and fitted to the experimental data (see Fig. S8). The best fit for each trabecula and temperature was determined by a global search of *hbz*, *d*, *n*_p_ and *n*_d_, and the intensity scaling factor *y* (Table S4), by minimizing χ^2^ calculated using the experimental standard deviations (see Fig. S8, shaded gray) from the difference between the experimental mean (black) and the model output (green) for the reciprocal space region 0.064–0.073 nm^−1^, corresponding to 70 detector pixels.

The best-fit to the M2L, M2H, and M6 meridional intensity distributions at 39°C in intact trabeculae was calculated using a similar approach, but adjusting the maximum number of point-diffractors *n*_d_ in each half-filament and the axial periodicity *d*.

### Online supplemental material

**Figure S4.**
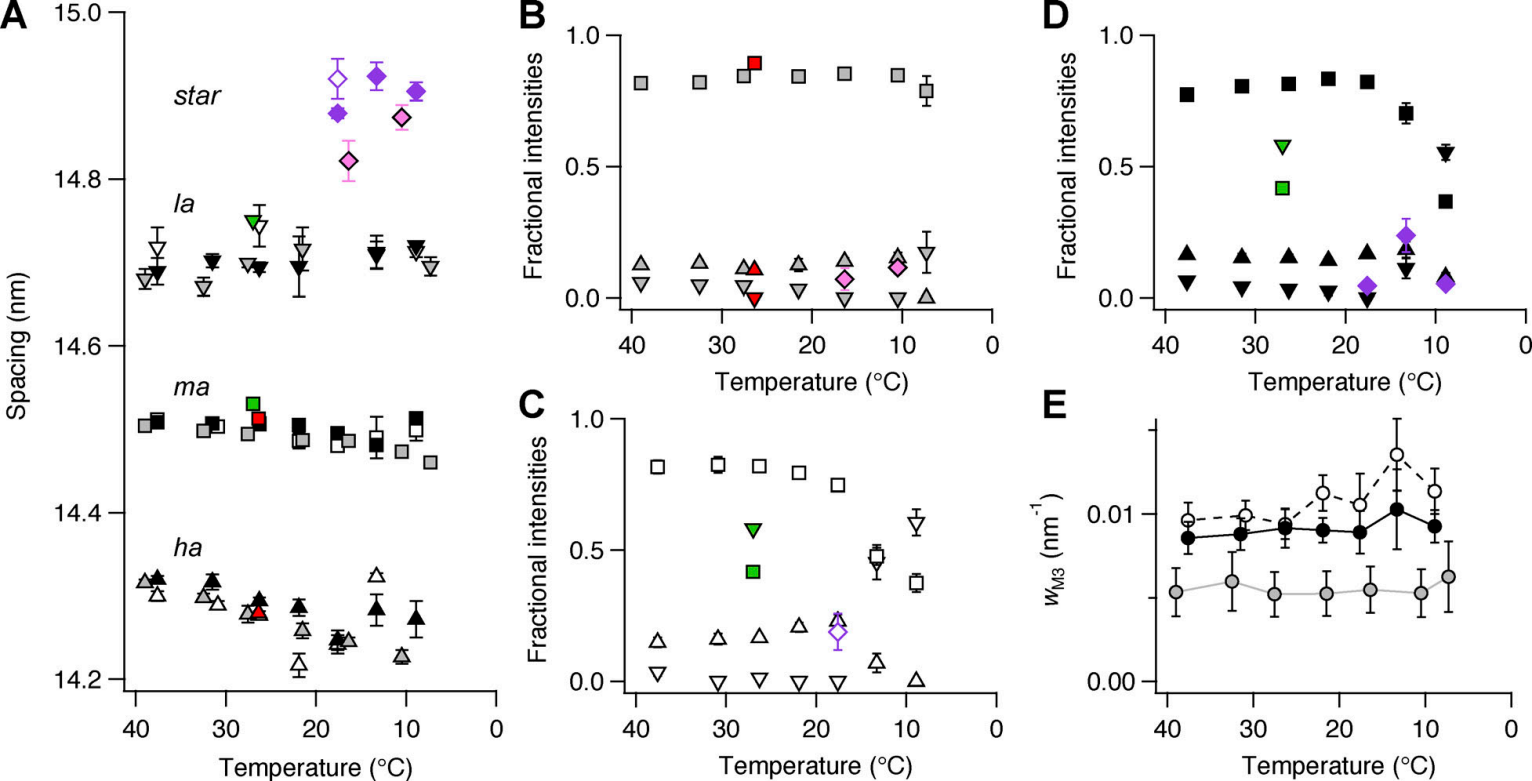
**Effect of cooling on the component peaks of the M3 reflection and the star peak. (A–D)** Spacing (A) and fractional intensity in intact (B) and demembranated trabeculae in the absence (C) and presence (D) of Dextran. Diamonds, star peak; inverted triangles, *la* peak; squares, *ma* peak; triangles, *ha* peak. Gray and pink symbols, intact trabeculae; black and purple symbols, demembranated trabeculae in the presence (filled symbols) or in the absence (empty symbols) of Dextran. Mean ± SE; *n* = 5 intact trabeculae and *n* = 4 demembranated trabeculae. Red symbols in A and B, diastolic value in intact trabeculae paced at 1 Hz; T = 26.4°C; data added from *n* = 6 trabeculae. Green symbols in A, C, and D, demembranated trabeculae in the presence of 3% Dextran at full calcium activation, pCa 4.7; T = 27°C; data from Brunello et al., 2020. **(E)** Cross-meridional width of the M3 reflection (*w*_M3_). Gray symbols, intact trabeculae; black symbols, demembranated trabeculae in the presence (filled symbols) or absence (empty symbols) of Dextran. Mean ± SE; *n* = 5 intact trabeculae and *n* = 4 demembranated trabeculae.

**Figure S5.**
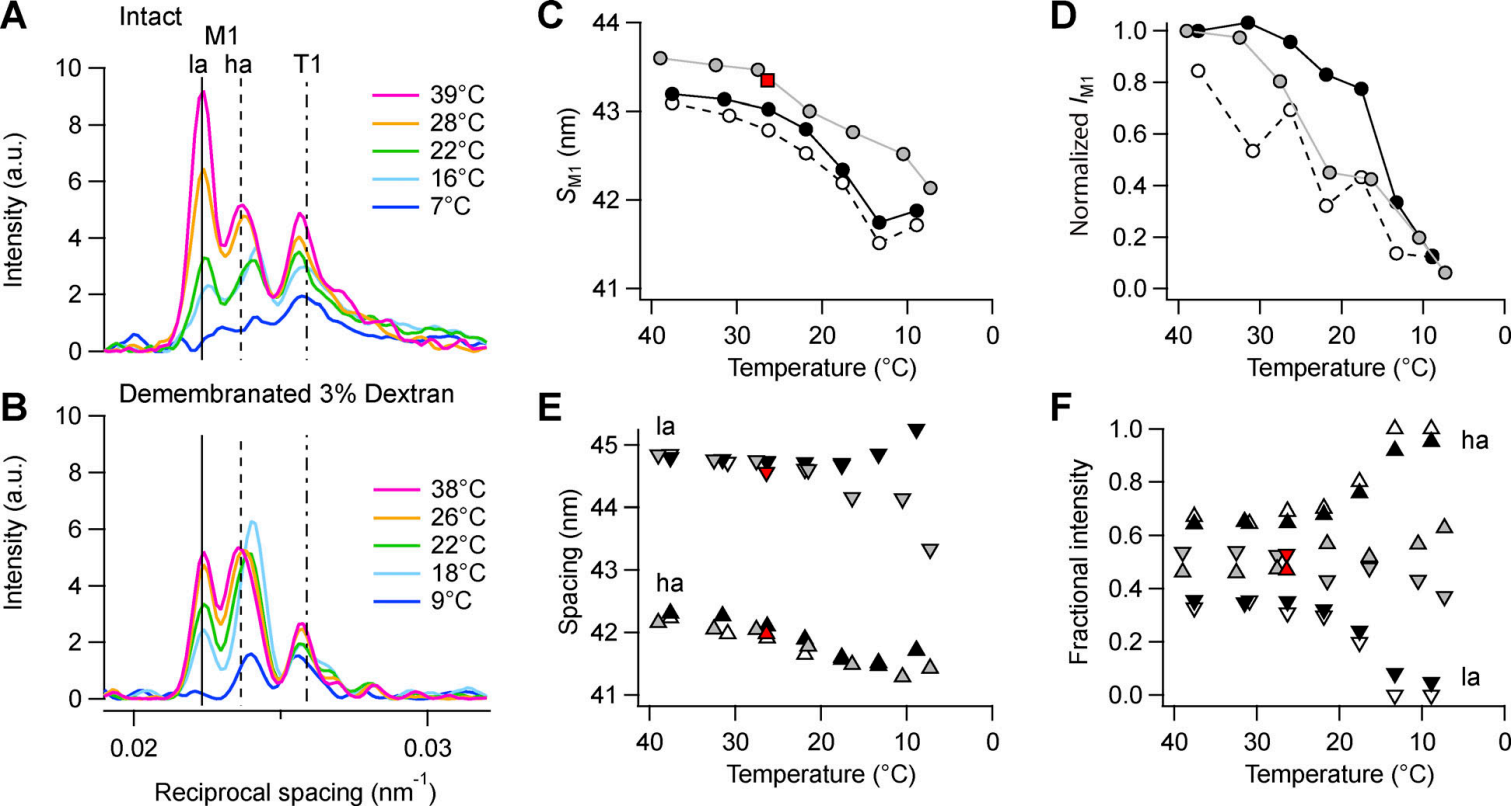
**Effect of cooling on the M1 reflection. (A and B)** Axial profiles of the M1 and T1 reflections from intact trabeculae (A) and demembranated trabeculae in the presence of dextran (B). Magenta, 39/38°C; orange, 28/26°C; green, 22°C; cyan, 16/18°C; blue, 7/9°C in intact and demembranated trabeculae, respectively. Vertical continuous and dashed lines, position of the two main peaks of the M1 reflection, and dashed-dotted line, average position of the T1 reflection in intact trabeculae at 39°C. **(C–F)** M1 spacing (*S*_M1_; C), total intensity (*I*_M1_; D), and spacing (E) and fractional intensity (F) of its two component peaks (*ha*, triangles; *la*, inverted triangles). Data added from *n* = 5 intact trabeculae and *n* = 4 demembranated trabeculae. Gray symbols, intact trabeculae; black symbols, demembranated trabeculae in the presence (filled symbols) or absence (empty symbols) of Dextran. Red symbols, diastolic values in intact trabeculae paced at 1 Hz; T = 26.4°C; data added from *n* = 6 trabeculae. Data in D normalized by the value at 39/38°C in intact trabeculae and in demembranated trabeculae in the presence of Dextran. a.u., arbitrary unit.

**Figure S6.**
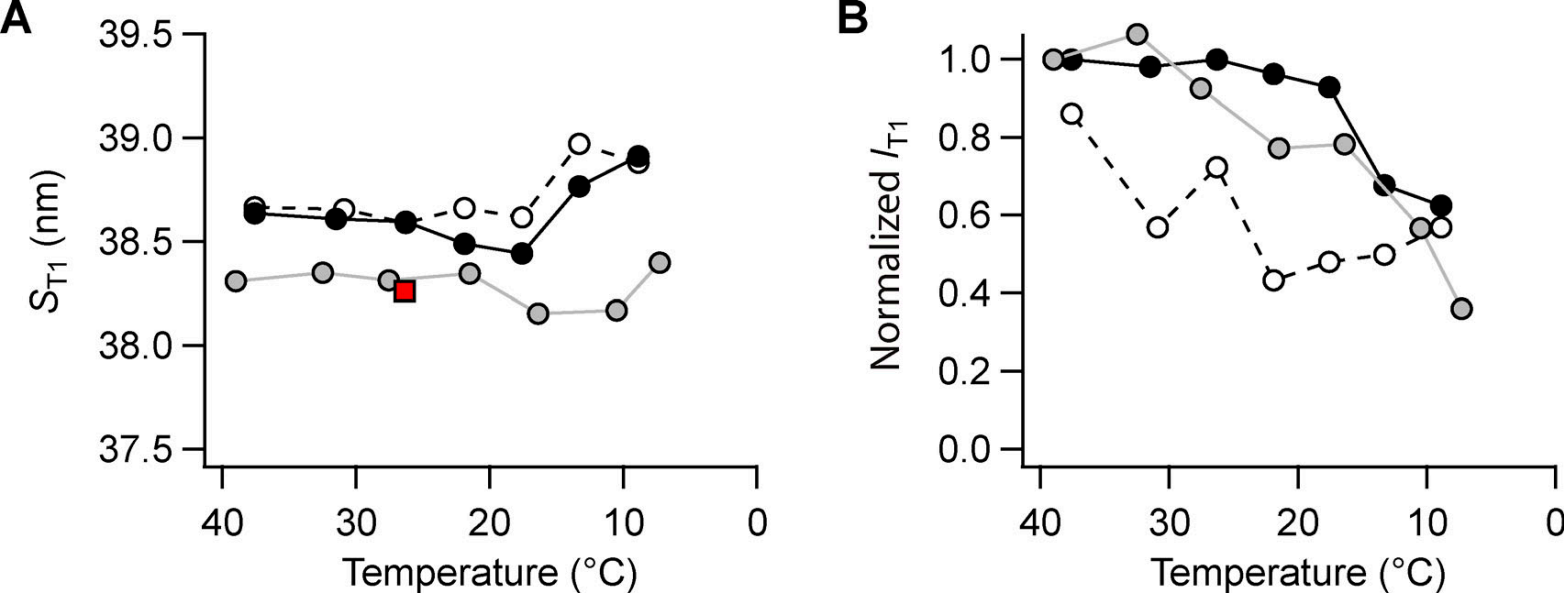
**Effect of cooling on the T1 reflection. (A and B) **T1 spacing (*S*_T1_; A), total intensity (*I*_T1_; B). Gray symbols, intact trabeculae; black symbols, demembranated trabeculae in the presence (filled symbols) or absence (empty symbols) of Dextran. Data added *n* = 5 intact and *n* = 4 demembranated trabeculae. Red square in A, diastolic value in intact trabeculae paced at 1 Hz; T = 26.4°C; data added from *n* = 6 trabeculae. Intensities normalized by the value at 39/38°C in intact trabeculae and in demembranated trabeculae in the presence of Dextran.

**Figure S7.**
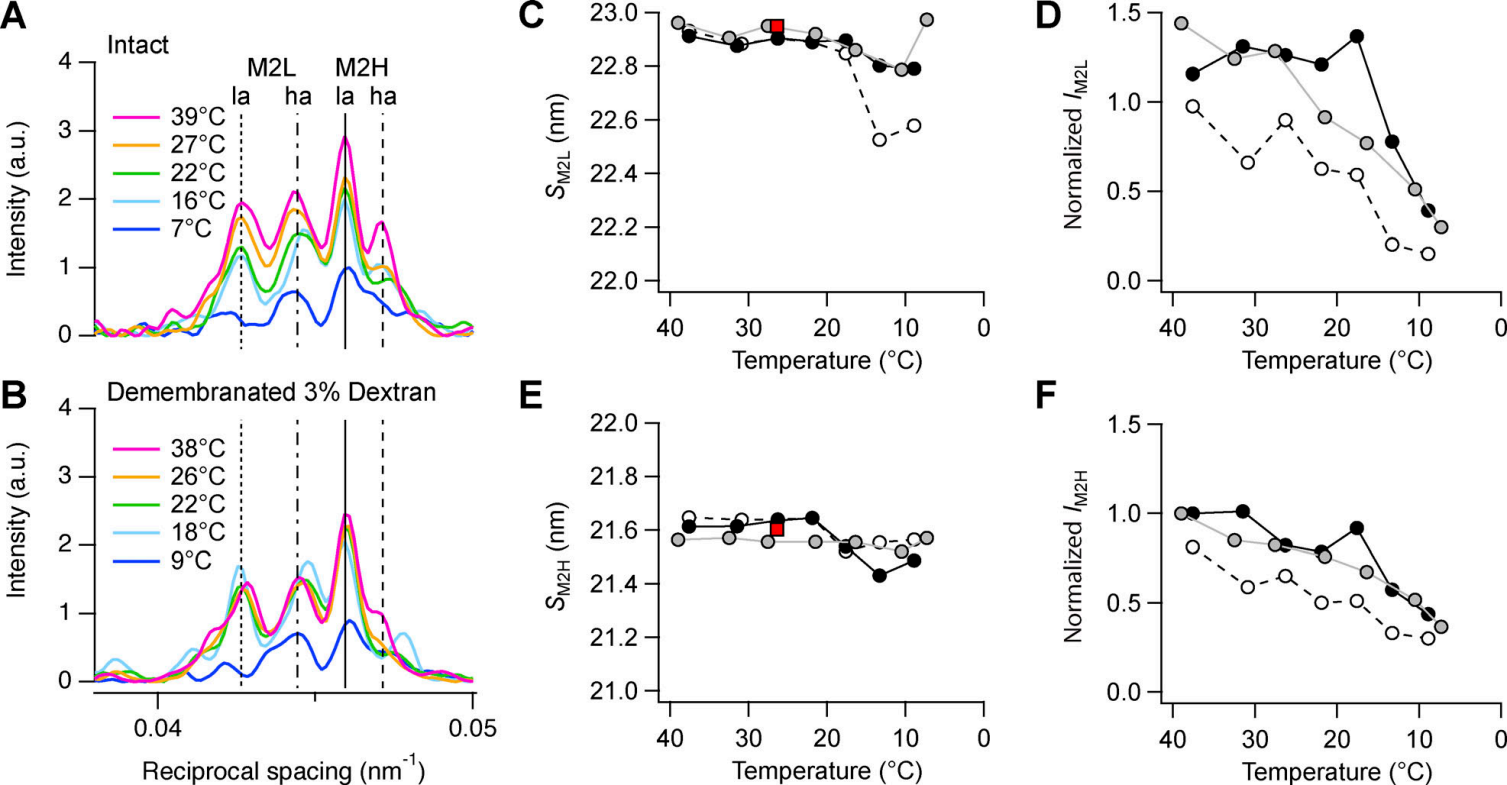
**Effect of cooling on the M2 reflection. (A and B)** Axial profiles of the M2 reflection from intact trabeculae (A) and demembranated trabeculae in the presence of Dextran (B). Magenta, 39/38°C; orange, 27/26°C; green, 22°C; cyan, 16/18°C; blue, 9/7°C in intact and demembranated trabeculae, respectively. Vertical dotted and dashed-dotted line, position of the two main peaks of M2L; continuous and dashed lines, position of the two main peaks of M2H in intact trabeculae at 39°C. **(C–F)** M2L spacing (*S*_M2L_; C) and total intensity (*I*_M2L_; D) and M2H spacing (E) and total intensity (F). Gray circles and line, intact trabeculae; empty circles and dashed black line, demembranated trabeculae no Dextran; black circles and continuous line, demembranated trabeculae in the presence of Dextran. Data added from *n* = 5 intact trabeculae and *n* = 4 demembranated trabeculae. Red square in C and E, diastolic value in intact trabeculae paced at 1 Hz; T = 26.4°C; data added from *n* = 6 trabeculae. Intensities in D and F are normalized by *I*_M2H_ at 39/38°C in intact trabeculae and in demembranated trabeculae in the presence of Dextran. a.u., arbitrary unit.

**Figure S8.**
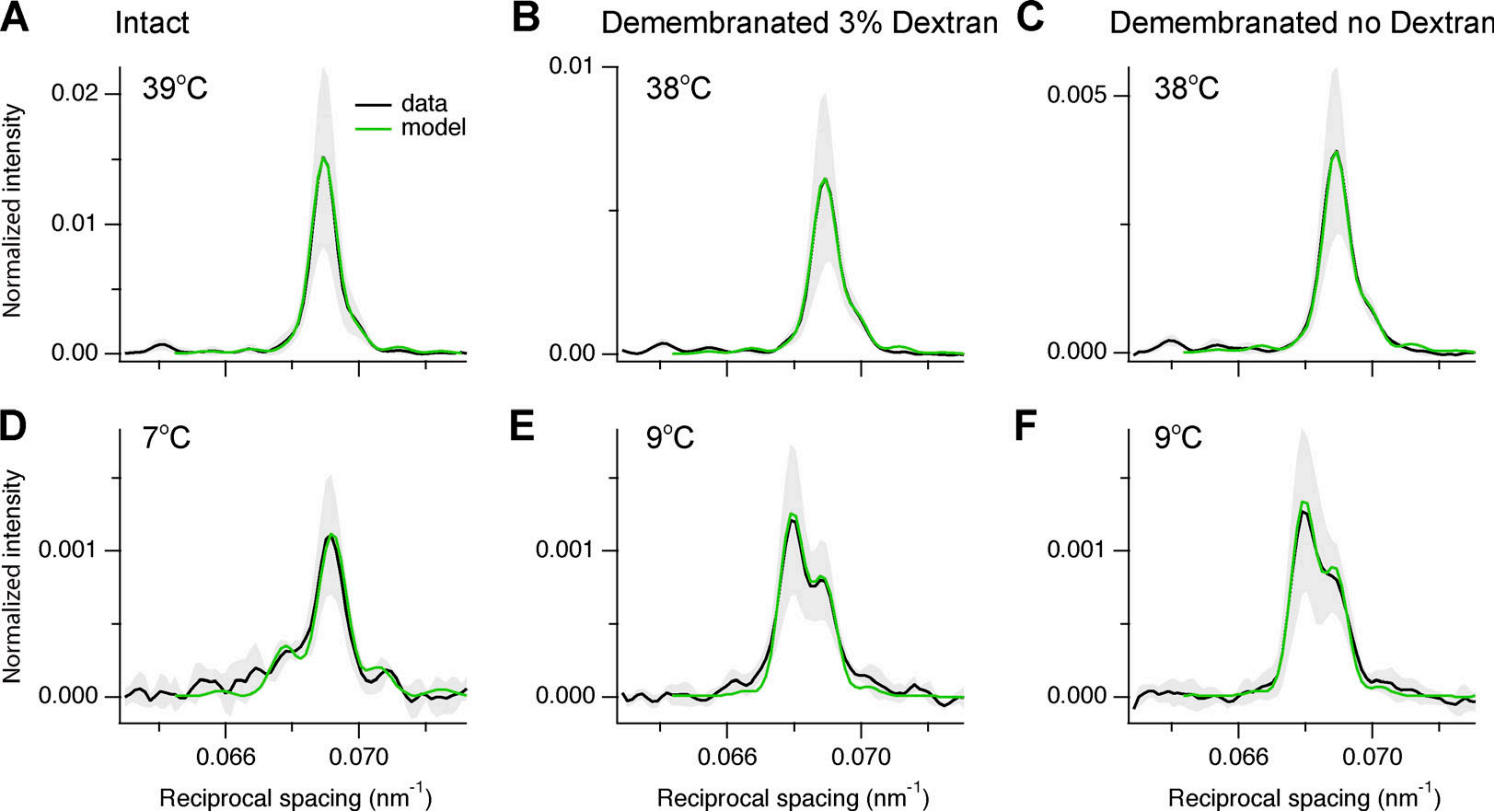
**Fitting the axial profile of the M3 reflection with a structural model of the thick filament.** **(A–F)** Experimental axial profiles (black) of the M3 reflection from intact trabeculae (A and D), demembranated trabeculae in the presence (B and E) and absence (C and F) of 3% Dextran with superimposed best fits from the model (green). Temperature, 39–38°C (A–C) and 7–9°C (D–F). Data are mean ± SD (grey shading) and were averaged from *n* = 4 trabeculae after normalization for *I*_10_ at 39°C and 38°C in intact (A and D) and in demembranated trabeculae in the presence of Dextran (B and C and E and F), respectively.

Fig. S1 shows equatorial and meridional intensity distributions from the same cardiac trabecula either quiescent or in the diastolic phase between beats. Fig. S2 shows reversibility of the effect of temperature on the structure of the thick filament in one intact and one demembranated cardiac trabecula. Fig. S3 shows polarized fluorescence measurements of the effect of changes in pH on myosin motor conformation. Fig. S4 shows the effect of cooling on the M3 sub-peaks and the star peak. Fig. S5 shows the effect of cooling on the M1 reflection. Fig. S6 shows the effect of cooling on the T1 reflection. Fig. S7 shows the effect of cooling on the M2 reflection. Fig. S8 shows the axial profile of the M3 reflections and their fitting with a structural model of the myosin filament. Table S1 reports the spacing of the myosin-based reflections of intact and demembranated cardiac trabeculae at physiological temperature. Table S2 reports comparison of x-ray parameters at 39°C and 7°C in intact and demembranated trabeculae. Table S3 reports the transition temperatures of myosin-based x-ray signals. Table S4 reports the model parameters for the best-fit of the axial profile of the myosin reflections.

## Results

### X-ray diffraction patterns from quiescent intact and relaxed demembranated trabeculae

X-ray diffraction patterns from intact quiescent trabeculae isolated from the right ventricle of rat hearts were collected at 39°C and SL 2.07 ± 0.05 µm (mean ± SD, *n* = 4 trabeculae; Fig. 1 A). The horizontal axis of the pattern (equator) contains a series of reflections originating from the hexagonal lattice of myosin and actin filaments and indexed as the lattice planes (Haselgrove and Huxley, 1973). The brightest equatorial reflections are the 1,0 and 1,1. The quasi-helical arrangement of the myosin motors in the thick filaments generates off-axis layer line reflections, of which the strongest is ML1, corresponding to an axial periodicity of ∼43 nm (Huxley and Brown, 1967). The meridional M3 reflection is associated with the axial periodicity of the myosin motors (14.48 nm), whereas the M6 reflection, with periodicity 7.24 nm, is primarily associated with the thick filament backbone (Reconditi et al., 2004; Huxley et al., 2006). Other myosin-based reflections (M1, M2, M4, M5), called “forbidden reflections,” index partly on the fundamental myosin periodicity of 43 nm and partly on a slightly longer periodicity and are associated with axial perturbations within those periodicities (Huxley and Brown, 1967; Fig. 1 F, magenta). The higher-angle (H) sub-peaks (Fig. 1 F, horizontal continuous lines) index on the ∼43-nm helical periodicity, whereas the lower-angle (L) sub-peaks (Fig. 1 F, horizontal dashed lines) index on a ∼45-nm axial periodicity that has been associated with myosin-binding protein-C (MyBP-C; Oshima et al., 2007; Oshima et al., 2012) or the C-type titin repeats (Caremani et al., 2021). The T1 reflection is associated with the axial periodicity of troponin in the thin filament (∼38 nm). All the meridional reflections show finely spaced sub-peaks due to sampling by interference fringes generated by the centrosymmetric structure of the thick filament (Fig. 1 E; Linari et al., 2000).

The x-ray diffraction pattern from intact quiescent trabeculae at 27°C is similar to that from electrically paced trabeculae in the relaxed or diastolic phase between beats (Fig. S1), and that from quiescent trabeculae at 39°C was similar to that from relaxed demembranated trabeculae in the presence of 3% Dextran at 38°C (SL 2.12 ± 0.02 µm; mean ± SD, *n* = 4 trabeculae; Fig. 1 B). The meridional reflections from intact and demembranated trabeculae at 38–39°C had generally similar spacings, intensities, and fine structure (Fig. 1 F and Table S1), with the exception of the M1 reflection, in which the two component peaks had similar spacings (Table S1) but different relative intensities (Fig. 1 F).

Cooling to 7–9°C induced an overall decrease in the intensity of all the x-ray reflections, in both quiescent intact (Fig. 1, C and G) and relaxed demembranated trabeculae (Fig. 1, D and G). The following sections describe the changes in individual reflections induced by cooling intact quiescent and relaxed demembranated trabeculae in the absence or presence of 3% Dextran T500 and compare them with those induced by electrical stimulation and maximal calcium activation in intact and demembranated trabeculae at 27°C, respectively.

### Equatorial reflections

Cooling intact quiescent trabeculae led to a decrease in the intensity of the equatorial reflections (Fig. 2, A and E), and they moved to a higher angle, indicating a decrease in the spacing between the 1,0 lattice planes containing the myosin filaments (*d*_10_; Fig. 2 C, gray circles). *d*_10_ was 35.4 ± 0.3 nm (mean ± SE, *n* = 5 trabeculae) at 39°C and decreased slightly on cooling to 7°C, to 34.1 ± 0.3 nm (Table S2). The ratio of the intensities of the *I*_11_ and *I*_10_ reflections (*I*_11_/*I*_10_), often used as an indicator of the movement of mass within the myofilament lattice, increased on cooling from 0.26 ± 0.01 at 39°C to 0.42 ± 0.05 at 7°C (Fig. 2 D and Table S2). This increase is much smaller than that associated with electrical stimulation at 27°C (Fig. 2 D, gray square [diastole]; gray diamond [systole]; Brunello et al., 2020). The radial width of the 1,0 and 1,1 reflections increased on cooling, indicating increased disorder of the myofilament lattice (Fig. 2 A and Table S2; Yu et al., 1985).

Demembranation of the trabeculae increased *d*_10_ to 38.9 ± 0.7 nm at 38°C (Fig. 2 C, empty circles), with little change in *I*_11_/*I*_10_ (Fig. 2 D, empty circles). The radial width of the 1,0 reflection (*w*_10_) increased slightly (Fig. 2, A and B; and Table S2). In contrast with the results for intact quiescent trabeculae, *d*_10_ increased to 41.7 ± 0.4 nm on cooling demembranated trabeculae to 9°C. *I*_10_ decreased on cooling (Fig. 2 F, empty circles), whereas *I*_11_ (empty triangles) and *I*_11_/*I*_10_ (Fig. 2 D, empty circles) increased. The effect of cooling on *I*_11_/*I*_10_ was larger than that in intact trabeculae. Cooling had little effect on *w*_10_ in demembranated trabeculae (Fig. 2 B and Table S2).

Addition of 3% Dextran T500 to demembranated trabeculae at 38°C compressed the myofilament lattice so that *d*_10_ became 35.1 ± 0.4 nm (Fig. 2C, black circles), similar to its value in intact quiescent trabeculae (gray circles), but *w*_10_ did not recover its value in intact trabeculae (Table S2). Cooling in the presence of Dextran induced an increase in *d*_10_ similar to that observed in its absence. *I*_11_/*I*_10_ (Fig. 2 D, black circles) increased to 0.91 ± 0.09 at 9°C, similar to the value at peak force in intact electrically paced trabeculae at 27°C (gray diamond), but lower than that at maximal calcium activation (black diamond; Brunello et al., 2020).

### Myosin layer lines

The intensity of the ML1 reflection (*I*_ML1_) from the folded and helically ordered myosin motors on the surface of the thick filament decreased on cooling in both intact quiescent trabeculae (Fig. 3, A and C, gray circles) and demembranated trabeculae, in both the presence (Fig. 3, B and C, black circles) and absence (Fig. 3 C, empty circles) of Dextran. *I*_ML1_ increased on the addition of Dextran at 38°C (Fig. 3 C, black circles; and Table S2). The dependence of *I*_ML1_ on temperature was roughly sigmoidal with a transition temperature (*T*_0.5_), the temperature at which the intensity changes were half-maximal, of ∼19°C in intact trabeculae and demembranated trabeculae in the presence of Dextran and of ∼24°C in the absence of Dextran (Table S3). At 7–9°C, *I*_ML1_ was reduced to <10% of its value at physiological temperature, which contrasts with the more modest reduction to 30–35% at peak systolic force in electrically paced trabeculae (Brunello et al., 2020).

### M3 reflection

The axial profile of the M3 reflection in intact quiescent trabeculae at 39°C is characterized by a dominant central or mid-angle (*ma*) peak and small low-angle (*la*) and high-angle (*ha*) satellites (Fig. 4 A, magenta), similar to the profiles observed in resting intact skeletal muscles (Linari et al., 2000; Reconditi et al., 2011; Caremani et al., 2019) and in the diastolic phase in electrically paced trabeculae at 27°C (Reconditi et al., 2017; Brunello et al., 2020). This intensity distribution arises from the centrosymmetric structure of the myosin filament, which generates interference fringes corresponding to the distance between the centers of the two diffracting arrays of motors in each filament *ID* (Fig. 1 E). These fringes sample the x-ray reflection that would be produced by an individual array of motors, producing the closely spaced sub-peaks. The total integrated intensity of the M3 reflection (*I*_M3_) in intact trabeculae decreased markedly on cooling (Fig. 4 A), with a transition temperature of ∼18°C (Fig. 4 D, gray; and Table S3). The spacing of the M3 reflection (*S*_M3_; Fig. 4 C, gray circles) was 14.490 ± 0.003 nm at 39°C, decreased upon cooling to a minimum of 14.435 ± 0.006 nm at 11°C, then recovered slightly on further cooling to 7°C (Table S2) to a value similar to that at physiological temperature. At 27°C, *S*_M3_ had a similar value in the quiescent state and in the diastolic state in electrically paced trabeculae (Fig. 4 C, gray square). The *la* peak of the M3 reflection was not detectable at 16°C or 11°C, but a new, even lower-angle, peak appeared with spacing ∼14.85 nm (Fig. 4 A, star peak; and Fig. S4, A and B, pink diamonds). This peak is probably due to interference involving a structural component with a slightly longer periodicity (Caremani et al., 2021).

Demembranation did not affect the spacing of the M3 reflection (Fig. 4 C; and Table S2) or that of its component peaks at 38°C (Fig. S4 A, empty symbols), but its cross-meridional width nearly doubled (Fig. S4 E, empty symbols), indicating greater axial misalignment between neighboring myosin filaments (Huxley et al., 1982). In demembranated trabeculae, *I*_M3_ decreased on cooling with a transition temperature of ∼25°C (Table S3), although the effect was truncated at the lowest temperatures studied, where *I*_M3_ retained 30–40% of its value at 38°C (Fig. 4 D). *S*_M3_ also decreased with a minimum at 18°C (Fig. 4 C), then increased steeply at lower temperatures, reaching 14.626 ± 0.010 nm at 9°C, close to its value during maximal calcium activation (Fig. 4 C, black diamond; and Table S2). The large increase in *S*_M3_ at low temperature in demembranated trabeculae was accompanied by an increase in the relative intensity of the *la* peak and a decrease in that of the *ma* peak (Fig. S4 C, empty symbols), as observed during maximal calcium activation at 27°C (Fig. 4 B, gray trace; and Fig. S4 C, green symbols).

Addition of 3% Dextran had no effect on *S*_M3_ at 38°C (Fig. 4 C) or on the relative intensity and spacing of its component peaks (Fig. S4, A and D, black symbols), but increased *I*_M3_ by ∼20% (Fig. 4 D, black circles). The transition temperature decreased to 19°C, similar to the value observed in intact trabeculae (Table S3). The cross-meridional width of the M3 reflection decreased slightly, but did not recover its value in intact trabeculae (Fig. S4 E, black circles). After reaching a minimum at 13°C, *S*_M3_ increased steeply (Fig. 4 C), accompanied by an increase in the relative intensity of the *la* peak and a decrease in that of the *ma* peak (Fig. S4 D). *S*_M3_ and the profile of the M3 reflection were similar at 9°C and at the plateau of the isometric contraction during maximal calcium activation at 27°C (Fig. 4 B). In the presence of Dextran, the star peak was present at all temperatures below 20°C (Fig. S4, A and D, purple diamonds).

### M6 reflection

The axial profile of the M6 reflection in intact trabeculae at 39°C has two sub-peaks of similar intensity (Fig. 5 A, magenta trace, *la* and *ha* peaks). Its total intensity (*I*_M6_) decreased on cooling to 11°C, (Fig. 5, A and D), accompanied by nearly doubling of its cross-meridional width (Table S2). Its spacing (*S*_M6_) at 39°C was 7.24 nm, roughly half of *S*_M3_ in those conditions (Table S1), and increased on cooling with a transition temperature of ∼12°C (Fig. 5 C and Table S3). The intensity of the *ha* peak was selectively reduced by cooling to 28°C (Fig. 5 A, orange trace), but both peaks became weaker and shifted to higher spacing at lower temperatures (Fig. 5 A, cyan and blue traces; and Fig. 5 C, gray circles). At 7°C, *S*_M6_ was 7.32 ± 0.02 nm, similar to its value at peak force in electrically paced intact trabeculae at 27°C (Fig. 5 C, gray diamond; Brunello et al., 2020).

Demembranation did not affect *S*_M6_ at 38°C, and neither did lattice compression by Dextran (Fig. 5 C; and Table S2). *I*_M6_ was slightly larger in the presence of Dextran, but was relatively insensitive to cooling (Fig. 5 D). *S*_M6_ increased on cooling with a transition temperature of ∼18°C in the absence of Dextran (Fig. 5 C, empty circles; and Table S3) and ∼15°C in its presence (Fig. 5 C, black circles; and Table S3). *S*_M6_ at 9°C was similar to that measured at peak force in electrically paced trabeculae at 27°C (Fig. 5 C, gray diamond; Brunello et al., 2020).

### Forbidden reflections

The forbidden reflections are associated with a systematic axial perturbation within the fundamental myosin periodicity of 43 nm (Huxley and Brown, 1967) and a slightly higher periodicity of unknown origin (Caremani et al., 2021). The M1 and M2 reflections could be characterized with high signal-to-noise (Fig. 1). In intact trabeculae at 39°C, the M1 reflection is composed of two main peaks, with the *la* peak more intense than the *ha* peak (Fig. S5 A, magenta; and Fig. S5 F, gray symbols), clearly separated from the T1 reflection on the higher-angle side (Fig. S5 A and Fig. S6). The overall intensity of the M1 reflection (*I*_M1_) decreased on cooling with a transition temperature of ∼20°C (Table S3), and below 20°C the *ha* peak became dominant (Fig. S5 A, cyan and blue; and Fig. S5 F, gray symbols). In contrast with *S*_M6_, the spacing *S*_M1_ decreased on cooling, as both sub-peaks moved toward higher angle (Fig. S5, C and E, gray symbols; and Table S2). At 27°C, *S*_M1_ and the spacing and fractional intensities of the M1 sub-peaks had similar values in the quiescent state and in the diastolic state in electrically paced trabeculae (Fig. S5, C, E, and F, red symbols).

Demembranation had little effect on *S*_M1_ at 38°C, and neither did lattice compression by Dextran (Fig. S5, C and E; and Table S2). However, in demembranated trabeculae the *ha* peak was the more intense (Fig. S5 F, black and empty symbols). Cooling led to a decrease of *I*_M1_ with transition temperatures ∼15°C and 24°C in the presence and absence of Dextran, respectively (Table S3). Below 20°C the intensity of the *la* peak decreased, and it disappeared at the lowest temperatures (Fig. S5 B, blue; and Fig. S5 F).

The M2 reflection from intact trabeculae at 39°C is composed of four main peaks plus a small shoulder on the lower-angle side (Fig. S7 A, magenta). The two *ha* peaks indexed on the fundamental myosin periodicity of ∼43 nm (M2H; Table S1), while the two *la* peaks indexed on a slightly longer periodicity of ∼45.8 nm (M2L; Table S1). The intensities *I*_M2L_ and *I*_M2H_ decreased on cooling (Fig. S7 A, blue; and Fig. S7, D and F, gray), but their spacings *S*_M2L_ and *S*_M2H_ were almost constant (Fig. S7, C and E). However, the analysis of the M2H reflection may be affected by the contribution of a collagen ring with spacing ∼21.5 nm (corresponding to the third order of a fundamental periodicity of ∼64.5 nm), which overlaps its *la* peak (Fig. 1, A–D).

Demembranation had little effect on *S*_M2L_ and *S*_M2H_ at 38°C, and neither did lattice compression by Dextran (Fig. S7, C and E; and Table S1). The intensities of both reflections were slightly lower in the absence of Dextran; however, they all decreased on cooling (Fig. S7, D and F).

### Modeling the axial profile of the M3 reflection

**Figure 6.**
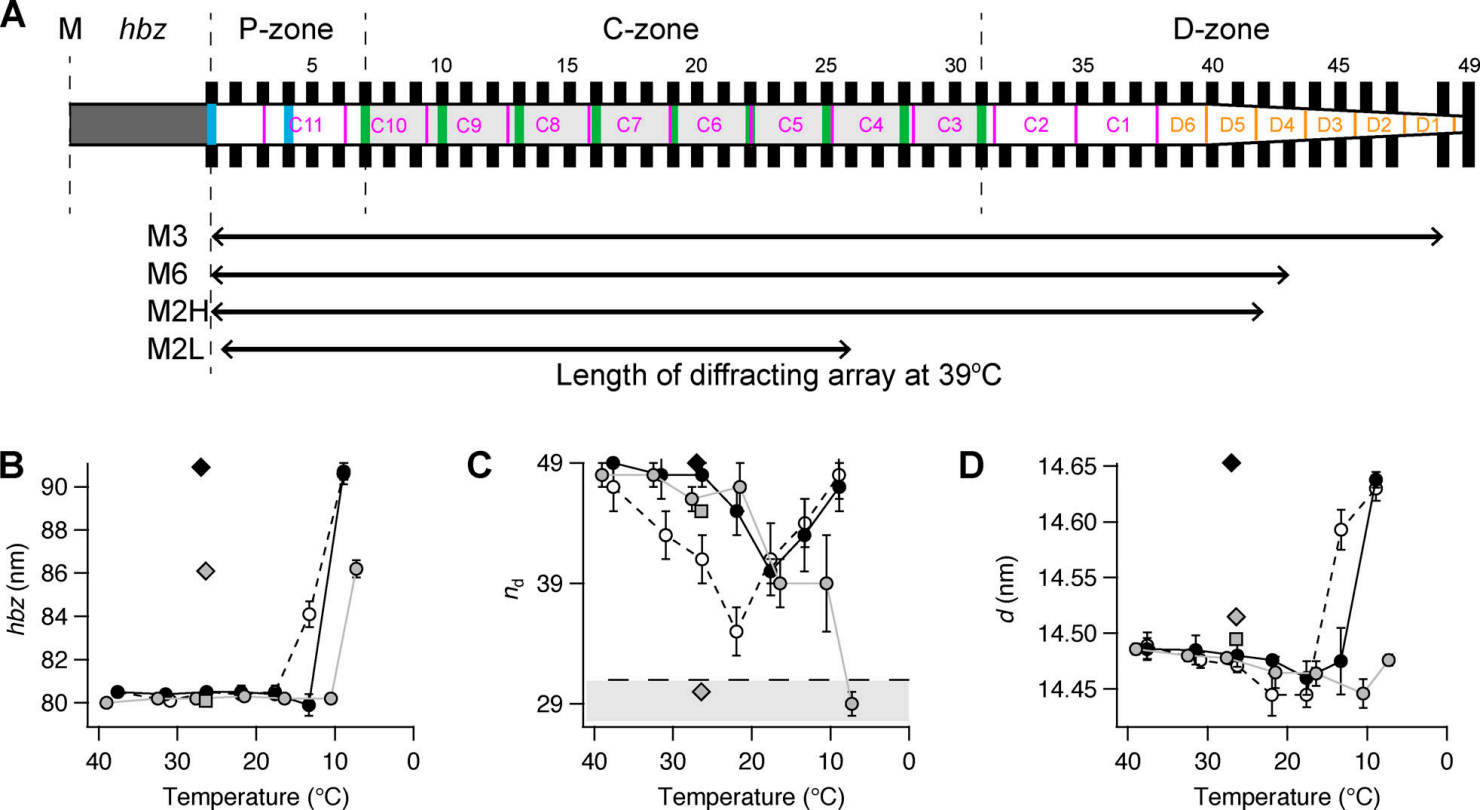
**Length and location on the half-thick filament of the diffracting arrays generating the myosin-based reflections. (A)** *hbz*, half-bare zone. P-, C-, and D-zones, proximal, MyBP-C containing, and distal zones. Black bars, layers of myosin head domains numbered 1–49 from the M line with average spacing of 14.49 nm. Green and blue vertical bars, MyBP-C and its paralogue proteins, respectively, assumed to align with “crown 1” of the myosin triplets in the C-zone (AL-Khayat et al, 2013). Magenta, titin C-type super-repeats C1–C11 numbered in the direction of the primary sequence. The distal end of C11 was placed 110 nm from the M line (Tonino et al., 2019); the proximal end of C1 is then [110 + (11 * 45.875)] = 614.6 nm from the M line, assuming an L periodicity of 45.875 nm. Orange, titin D-type super-repeats 1–6. Lower arrows indicate the length and position of the arrays of diffractors generating each myosin reflection estimated by x-ray interference (see main text). **(B–D)** Best-fit model parameters for the axial profile of the M3 reflection. *hbz* (B); distal layer marking the end of the region of ordered myosin motors, *n*_d_ (C); axial periodicity between adjacent layers of myosin motors, *d* (D). Light gray shading in panel C indicates C-zone with boundary at myosin layer 31, horizontal dashed line. Gray circles and line, intact trabeculae; empty circles and dashed black line, demembranated trabeculae in the absence of Dextran; black circles and continuous line, demembranated trabeculae in the presence of 3% Dextran. Mean ± SE; *n* = 5 intact trabeculae and *n* = 4 demembranated trabeculae. Gray square and diamond, diastolic and systolic peak force values, respectively, in intact trabeculae paced at 1 Hz (T = 26.4°C; data from Brunello et al. [2020]); black diamond, maximal calcium activation ([Ca^2+^] = 20 µM; 3% Dextran; T = 27°C; data from Brunello et al. [2020]).

The M3 axial profile and its separation into sub-peaks contain information about the length and location of the array of myosin motors contributing to the reflection and their average conformation (Linari et al., 2000; Brunello et al., 2020; Caremani et al., 2021; Hill et al., 2021). The multi-peak meridional intensity distribution (Fig. 1, F and G; and Fig. 4 A) is the product of the profile generated by an array of myosin motors on one side of the M-line and the fringe pattern generated by the two arrays of motors in each centrosymmetric thick filament with center-to-center or interference distance (*ID*; Fig. 1 E). Fig. 6 shows a schematic of the half-thick filament at physiological temperature, with 49 layers of myosin motors starting at ∼80 nm (*hbz*) from the M-line and average periodicity 14.49 nm (*d*). The MyBP-C containing C-zone spans from layer 7 to 31 (green bars and light gray shade on the thick filament), the proximal or P-zone layers 1 to 7, and the distal or D-zone layers 31 to 49 (Luther et al., 2008). The M3 axial profiles were reproduced using this distribution of diffractors in each thick filament (see Materials and methods and Fig. S8) by global fit of five free parameters: *hbz*, *d*, intensity scaling factor *y*, and first or proximal *n*_p_ and last or distal *n*_d_ layers of motors in a contiguous array (Table S4). Motors outside this array were considered to be isotropic and not to contribute to the reflection. The model was not able to reproduce the star peak observed below 20°C (Fig. 4, A and B; and Fig. S4 A), which likely originates from a longer periodicity on the thick filament axis, as in skeletal muscle (Caremani et al., 2021; Hill et al., 2021).

The best-fit to the axial profile of the M3 reflection in intact quiescent trabeculae at 39°C was obtained with nearly all the 49 layers of myosin motors in each half-filament contributing (*n*_p_ = 1 and *n*_d_ = 48; Fig. 6 C, Fig. S8 A, and Table S4). *n*_d_ decreased on cooling, whereas *n*_p_ increased slightly so that only the motors in layers 4–29 contributed to the M3 reflection at 7°C (Fig. 6 C and Table S4). Thus, at this temperature, the motors in the C-zone of the thick filament (Fig. 6) remain axially ordered, whereas those in the D-zone have become disordered. The center-of-mass of the ordered motors, reported by the parameter *hbz* (Fig. 6 B and Table S4), remained remarkably constant on cooling to 10°C, then moved ∼6 nm away from the M-line on further cooling to 7°C. This movement is similar to that during the rise of force in paced intact trabeculae at 27°C. Demembranation in either the presence or absence of Dextran at 38°C had little effect on either the filament location of the ordered motors (Fig. 6 C) or their center-of mass (*hbz*; Fig. 6 B). *n*_d_ decreased when demembranated trabeculae were cooled to 22°C in the absence of Dextran (Fig. 6 C, empty circles), but this effect was reversed on further cooling to 9°C. The biphasic effect of cooling on *n*_d_ was attenuated in the presence of 3% Dextran (black circles) so that its value at 22°C became similar to that in intact trabeculae (gray circle). The center-of-mass of the ordered motors moved ∼10 nm away from the M-line on cooling to 9°C (*hbz*; Fig. 6 B, empty and black circles), similar to the movement observed at maximum calcium activation at 27°C (black diamond). The best-fit values of the axial motor periodicity *d* (Fig. 6 D) were very similar to the experimental values of *S*_M3_ (Fig. 4 C).

A similar approach was used to calculate the filament location of the diffractors contributing to the M6, M2H, and M2L reflections at 39°C in intact trabeculae. The best fit to the M6 reflection was reproduced with *n*_p_ = 1 and *n*_d_ = 85 for a periodicity *d* = 7.24 nm (Table S4), corresponding to the region of the thick filament containing the first 43 layers of myosin motors (Fig. 6 A). The M2H sub-peaks were reproduced by an array of 26 point diffractors with periodicity *d* = 21.54 nm (Table S4), corresponding to roughly the first 42 layers of myosin motors (Fig. 6 A). The M2L sub-peaks were reproduced by interference between 16 point diffractors with periodicity *d* = 22.94 nm, corresponding to about eight repeats of the 45.9 nm L periodicity in each half-filament starting from the *hbz* (Fig. 6 A and Table S4).

## Discussion

### The structure of the thick filament at physiological temperature and filament lattice spacing

We used x-ray interference to determine the location in the thick filament of the periodic structures generating some of the meridional reflections (Linari et al., 2000; Brunello et al., 2020; Caremani et al., 2021; Hill et al., 2021). In intact quiescent trabeculae at 39°C, all 49 layers of myosin motors in each half-thick filament, from the edge of the bare zone to the filament tip, contribute to the M3 reflection (Fig. 6), with a periodicity that matches the 43-nm periodicity (H) of the myosin helix. The filament backbone periodicity that is primarily responsible for the M6 reflection comes from diffractors in the region of the half-thick filament containing the first 43 layers of myosin motors (Fig. 6 A), consistent with the idea that the M6 reflection arises from the region of the filament with a uniform cross section (i.e., excluding the tapering tip region). The axial perturbation that gives rise to the H component of the M2 reflection also extends along the uniform segment of the half-thick filament, excluding the tapering tip. The cardiac thick filament exhibits a second periodicity (L) that is ∼5% longer than the H periodicity and gives rise to the M2L sub-peaks. The length of the array of diffractors that generates this reflection roughly matches the C- and P-zones of the half-filament, which contain MyBP-C and its paralogue proteins (Fig. 6 A, green and blue bars, respectively), suggesting that the structure responsible for the L-periodicity reflections is localized in this region. Similar results were obtained in skeletal muscle (Malinchik and Lednev, 1992; Caremani et al., 2021).

Demembranation of cardiac trabeculae in relaxing conditions (pCa 9.0) leads to an increase in the filament lattice spacing (Irving et al., 2000). At 38°C, this lattice expansion is accompanied by increased lattice disorder and axial misalignment of neighboring thick filaments (Table S2). In contrast, the equatorial intensity ratio *I*_11_/*I*_10_, the axial periodicities of the filaments (Table S2), the average axial center-of-mass of the diffracting myosin motors as measured by *hbz* (Fig. 6 and Table S4), and the filament location of those motors do not change on demembranation. Although it was not possible to measure the change in diffracted intensities in the same preparation in the intact and demembranated states, these results suggest that, at 38°C, motors retain the folded helical conformation after demembranation. At the resolution of these x-ray measurements, thick filament structure at 38°C is not altered by the loss of soluble cytoplasmic proteins or possible changes in the phosphorylation levels of thick filament proteins associated with demembranation. Moreover, the structure is the same at pCa 9 in relaxing solution and at the roughly 100× higher calcium concentration in diastole (∼0.1 μM, pCa 7; Dibb et al., 2007).

At 38°C, addition of 3% Dextran to the relaxing solution restored the filament lattice spacing to that of intact trabeculae (Fig. 2 C and Table S2), although the increases in lattice disorder and axial misalignment of neighboring thick filaments observed on demembranation were not reversed by the addition of Dextran (Table S2). Lattice compression induced a ca 20% increase in the intensities of the myosin- (Fig. 4 D; Fig. 5 D; Fig. S5 D; and Fig. S7, D and F) and actin-based meridional reflections (Fig. S6 B). These general intensity increases may be explained by the increase in the number of myofilaments illuminated by the x-ray beam after lattice compression, but the increase in the intensity of the ML1 (Fig. 3 C) seems to be too large to be fully accounted for by this effect, as noted previously in skeletal muscle (Caremani et al., 2021). It seems likely, therefore, that compression of the filament lattice stabilizes the folded helical conformation of the myosin motors on the thick filament at 38°C. However, modeling of the axial profile of the M3 reflection (Fig. 6 and Table S4) showed that the average axial center-of-mass of the ordered motors and their filament location were not affected by lattice compression, indicating that, although the number of helical folded motors is increased by lattice compression, the mean conformation and filament location of the ordered motor population is unchanged.

### Effects of cooling intact quiescent trabeculae on thick filament structure

Cooling intact quiescent trabeculae to 7°C induced large decreases in the intensity of all the x-ray reflections (Fig. 1). The M3 and ML1 reflections in particular became very weak, signaling loss of the helical folded conformation of the myosin motors seen at physiological temperature. These changes are similar to those reported previously in skeletal muscle (Wray, 1987; Xu et al., 1997; Xu et al., 1999; Xu et al., 2003; Xu et al., 2006), where they were interpreted as a consequence of a temperature-dependent shift in the equilibrium of the myosin ATP hydrolysis step associated with a conformational change of the nucleotide-binding site of myosin from the “closed” M.ADP.Pi state to the “open” M.ATP state. These changes in motor conformation are associated with a slight expansion of the thick filament backbone, signaled by increases in *S*_M6_ and *S*_M3_, as also observed in skeletal muscle (Caremani et al., 2019; Caremani et al., 2021), although the behavior of *S*_M3_ is more complicated in both muscle types, as discussed further below.

Modeling of the M3 reflection showed that cooling selectively disorders the distal and proximal layers of myosin in the half-thick filament so that the myosin motors still contributing to the M3 reflection at 7°C are confined to the central region of the half-thick filament (layers 4–29 in Fig. 6, and Table S4). This corresponds approximately to the region containing MyBP-C, the C-zone (Fig. 6), suggesting that MyBP-C stabilizes the folded conformation of the myosin motors. Moreover, the average center of mass of the C-zone motors that stay ordered at low temperature was displaced ∼6 nm away from the M-line compared with that of the folded conformation (Fig. 6 B and Table S4), the same size as the displacement measured during contraction in electrically paced cardiac trabeculae at 27°C (Table S4; Brunello et al., 2020). The elongation of the filament backbone signaled by the spacing of the M6 reflection (*S*_M6_) is also similar in relaxing conditions at 7°C as at peak systolic force (Fig. 5). However, cooling to 7°C in quiescent conditions did not completely reproduce the filament structure and motor conformation seen at peak systolic force. The ML1 reflection remains more intense in the latter condition, indicating that about half of the motors remain in the helical folded conformation (Brunello et al., 2020), and the M3 reflection is much more intense, being dominated by the contribution of a small fraction of actin-bound force-generating motors in the C-zone (ibid). Thus, cooling quiescent trabeculae to 7°C reproduces some but not all of the structural features of systolic activation at 27°C.

The effects of cooling on the intensities of the ML1 layer line and the main meridional reflections were half-complete at a transition temperature of 18–20°C (Table S3), although the elongation of the filament backbone signaled by the spacing of the M6 reflection (*S*_M6_) had a lower transition temperature, ∼13°C. The axial periodicity of the myosin motors (*S*_M3_) had a distinct temperature dependence, first decreasing on cooling to a minimum at 10°C before recovering on further cooling. We return to the interpretation of this difference in a later section.

The interpretation of the effects of cooling intact quiescent trabeculae is complicated, however, by its effects on the intracellular ion concentrations. Cooling below 20°C inhibits the sodium-potassium pump with a consequent elevation of the intracellular sodium concentration and of calcium influx via the sodium-calcium exchanger (Shattock and Bers, 1987). Cardiac muscle cells become unexcitable in these conditions, and spontaneous sarcomere motion is observed (de Tombe and ter Keurs, 1990) due to spontaneous calcium release from the sarcoplasmic reticulum (Chiesi, 1979; Fabiato, 1985). To eliminate the possible contribution of these ionic changes to the cooling-induced changes in filament structure, it is important to characterize the structural changes in demembranated trabeculae, in which the ionic composition of the solution bathing the myofilaments can be controlled.

### Effects of cooling and filament lattice compression on thick filament structure in relaxed demembranated trabeculae

The effects of cooling from 38 to 7°C on myosin filament structure and motor conformation in demembranated relaxed trabeculae are similar in many respects to those in intact quiescent trabeculae. The helical folded conformation of the myosin motors, signaled by the intensity of the ML1 layer line (*I*_ML1_; Fig. 3), is almost completely lost on cooling, and the periodicity of the filament backbone, signaled by *S*_M6_ (Fig. 5), extends by the same amount as in intact quiescent trabeculae. These changes seem to be independent of the filament lattice spacing. Cooling of relaxed demembranated trabeculae in the absence of Dextran induced expansion of the interfilament lattice, perhaps associated with the increase in pH (see Materials and methods) and electrostatic repulsion between myofilaments (Millman and Irving, 1988; Millman, 1998), in contrast with the lattice compression observed on cooling in intact quiescent trabeculae (Fig. 2). Moreover, compression of the filament lattice by 3% Dextran had little effect on either *S*_M6_ or the residual *I*_ML1_ intensity at 7°C.

However, there are some quantitative differences between the effects of cooling in intact and demembranated trabeculae in addition to the lattice spacing changes described above. The equatorial intensity ratio *I*_11_/*I*_10_ increases more on cooling in demembranated trabeculae (Fig. 2), although this may be a secondary consequence of the expanded filament lattice. The *I*_M3_ is less affected by cooling in demembranated trabeculae, and its spacing (*S*_M3_) increases much more dramatically below 15°C (Fig. 4). Related to these differences, the average center-of-mass of the ordered motors (*hbz*; Fig. 6 B and Table S4) moves away from the M line by ∼10 nm in demembranated trabeculae at 9°C, independently of lattice compression by Dextran, compared with only ∼6 nm in intact trabeculae at 7°C. Moreover, the effect of cooling on the filament location of the ordered myosin motors, as signaled by the parameter *n*_d_, was characteristically different in intact and demembranated trabeculae. In both cases, D-zone motors became disordered on cooling to 15–20°C; however, on further cooling to 7–9°C, this effect was enhanced in intact trabeculae but reversed in demembranated trabeculae (Fig. 6 C). The difference may be related to the compressed lattice in intact trabeculae at low temperature or perhaps to the altered intracellular ion concentrations. The maintained order of D-zone motors in demembranated trabeculae at low temperature may be responsible for the increased axial periodicity of the motors (*S*_M3_) in these conditions, if the intrinsic axial periodicity of the filament is larger in the D-zone than the C-zone, as argued previously on the basis of the distinct changes in *S*_M3_ and *S*_M6_ following electrical stimulation of intact trabeculae at 27°C (Brunello et al., 2020). This hypothesis might also explain the distinct effects of cooling on the axial periodicity of the myosin motors (*S*_M3_, Fig. 4 C; *d*, Fig. 6 D) and the axial periodicity of the filament backbone (*S*_M6_, Fig. 5 C). The filament backbone elongates monotonically on cooling (*S*_M6_, Fig. 5 C), but the average periodicity of the myosin motors (*S*_M3_, Fig. 4 C; *d*, Fig. 6 D) does not follow this increase because, after cooling, a greater fraction of the ordered motors are in the C-zone, which has a slightly smaller axial periodicity.

The temperature dependence of the effects of cooling was markedly different in intact and demembranated trabeculae. The transition temperatures for the changes in the intensities of the ML1 layer line and the main meridional reflections and for *S*_M6_ were 4–7°C higher in demembranated than intact trabeculae (Table S3). This change was largely reversed when the filament lattice spacing of the demembranated trabeculae was restored to that of the intact trabeculae by addition of 3% Dextran. A similar Dextran-dependent shift in transition temperature was reported for the orientation of the myosin regulatory light chain measured using fluorescent probes in demembranated trabeculae (Park-Holohan et al., 2021). These results indicate that the folded helical conformation of myosin is stabilized by lattice compression, as previously reported for demembranated skeletal muscle fibers. This effect might be mediated by restoring MyBP-C links between thin and thick filaments by compression to the intact lattice spacing (Rahmanseresht et al., 2021).

### Optimal experimental conditions for investigating the control of contractility in ventricular trabeculae

Intact and demembranated trabeculae from rat heart have been used very extensively in the investigation of contractile and regulatory mechanisms in heart muscle. Most studies using electrically paced intact trabeculae have been conducted at ∼27°C in order to avoid the consequences of anoxia at higher temperatures, although more physiological temperatures can be employed if the trabeculae are exceptionally thin (Janssen et al., 2002). The results of the present experiments show that the structure of the myosin filaments and the conformation of the myosin motors in quiescent trabeculae at physiological temperature is almost completely preserved on cooling to 27°C. The intensities of the ML1 layer line (*I*_ML1_; Fig. 3) and of the M3 meridional reflection (*I*_M3_; Fig. 4) are ∼20% smaller at 27°C, but since diffracted intensities are proportional to the square of the number of diffractors, this corresponds to a reduction of only ca 10% in the number of folded helical motors. Filament and motor periodicities are also preserved in this temperature range, as are the average center-of-mass of the myosin motors (*hbz*; Fig. 6 and Table S4) and their filament location (*n_p_*, *n_d_*). Moreover, all these parameters are the same, within the resolution of the measurements, in quiescent trabeculae and in diastole in electrically paced trabeculae (Fig. S1). We conclude that for studies of contractile and regulatory mechanisms in trabeculae, the diastolic state in electrically paced trabeculae at 27°C is acceptably close to that at physiological temperature, although the kinetics of the changes on activation will, of course, be slower.

Demembranated trabeculae allow additional interventions in the study of contractile and regulatory mechanisms, most importantly in the control of the ionic composition of the solution bathing the filaments, especially its free calcium concentration, but also to modify the composition or the phosphorylation state of the filaments themselves. These advantages are achieved, however, at the potential cost of uncharacterized changes in the filaments and their environment, including the loss of function of many cytoplasmic and membrane-bound proteins. The present results show that, perhaps surprisingly, the structure of the myosin filaments and the conformation of the myosin motors at physiological temperature are almost completely preserved on demembranation. However, the structure is not preserved at the lower temperatures usually employed with this preparation, particularly in the absence of Dextran. For example, the intensity of the ML1 layer line, signaling the helical order of the motors and filaments at physiological temperature, decreases on cooling with a transition temperature of ∼24°C in the absence of Dextran (Table S3). Thus in the many previous studies of demembranated trabeculae performed at room temperature in the absence of Dextran, the helical order of the motors was not preserved in relaxing conditions. As in intact trabeculae, this problem can be largely overcome by conducting such studies at around 27°C in the presence of Dextran, which restores the transition temperature of the ML1 intensity to that in intact trabeculae and also preserves the order of the D-zone motors in relaxing conditions (Fig. 6 and Table S4).

## Supplementary Material

Table S1 reports the spacing (in nanometers) of the myosin-based layer lines and meridional reflections at 38–39°C in intact and demembranated trabeculae in the presence of 3% Dextran T500.

Table S2 lists x-ray parameters at 39°C and 7°C in intact quiescent and demembranated trabeculae in the absence and presence of Dextran.

Table S3 reports the transition temperatures of x-ray parameters.

Table S4 provides the model parameters for the best fit of the axial profile of the myosin (M) reflections.
