# Supplementary material for: Cooling intact and demembranated trabeculae from rat heart releases myosin motors from their inhibited conformation
Source: J Gen Physiol. 2022 Jan 28;154(3):e202113029. doi: 10.1085/jgp.202113029 (PMC8823665; doi:10.1085/jgp.202113029)
Supplement: Table S1 — reports the spacing (in nanometers) of the myosin-based layer lines and meridional reflections at 38–39°C in intact and demembranated trabeculae in the presence of 3% Dextran T500. [file JGP_202113029_TableS1.docx]

|  |  | **Intact** | **Expected *L* and *H*** | **Demembranated 3% Dex** |
| --- | --- | --- | --- | --- |
| *L* fundamental |  |  | 45.875 |  |
| *H* fundamental |  |  | 43.467 |  |
| M1 | *la* | 44.840 | 45.875 | 44.797 |
|  | *ha* | 42.157 | 43.467 | 42.311 |
| M2 | *L* | 22.962 | 22.937 | 22.913 |
|  | *H* | 21.564 | 21.734 | 21.613 |
| M3 | *L* | n/a | 15.292 | n/a |
|  | *H* | 14.490 (± 0.003) | 14.489 | 14.488 (± 0.005) |
| M6 | *L* | n/a | 7.646 | n/a |
|  | *H* | 7.240 (± 0.002) | 7.245 | 7.245 (± 0.005) |

**Table S1. Spacing (in nm) of the myosin-based layer lines and meridional reflections at 38-39°C in intact and demembranated trabeculae in the presence of 3% Dextran T500.** Main myosin periodicity H and longer periodicity L calculated as 3 and 2 times the average of the M3H and M2L spacings of intact and demembranated trabeculae in the presence of Dextran, respectively. Data are added or mean ± SE from n=5 intact and 4 demembranated trabeculae.
