# Supplementary material for: Cooling intact and demembranated trabeculae from rat heart releases myosin motors from their inhibited conformation
Source: J Gen Physiol. 2022 Jan 28;154(3):e202113029. doi: 10.1085/jgp.202113029 (PMC8823665; doi:10.1085/jgp.202113029)
Supplement: Table S2 — lists x-ray parameters at 39°C and 7°C in intact quiescent and demembranated trabeculae in the absence and presence of Dextran. [file JGP_202113029_TableS2.docx]

|  | **Intact** | | **Demembranated no Dex** | | **Demembranated 3% Dex** | |
| --- | --- | --- | --- | --- | --- | --- |
|  | **39°C** | **7°C** | **38°C** | **9°C** | **38°C** | **9°C** |
| ***d*_10_ (nm)** | 35.4 ± 0.3 | 34.1 ± 0.3  *(0.0244)* | 38.9 ± 0.7  *(*0.0084)* | 41.7 ± 0.4  *(0.0043)* | 35.1 ± 0.4  *(*^$^*0.5235)* | 38.0 ± 0.6  *(0.0017)* |
| ***I*_10_** | 1 | 0.32 ±0.07  *(0.0027)* | 1.43 ±0.06  *(*0.0063)* | 1.00 ± 0.02  *(0.0111)* | 1  *(*^$^*n/a)* | 0.73 ± 0.04  *(0.0059)* |
| ***I*_11_** | 0.26 ± 0.01 | 0.14 ± 0.04  *(0.0575)* | 0.33 ± 0.02  *(*0.2266)* | 0.76 ± 0.02  *(0.0009)* | 0.25 ± 0.02  *(*^$^*0.5848)* | 0.65 ± 0.04  *(0.0011)* |
| ***I*_11_/*I*_10_** | 0.26 ± 0.01 | 0.42 ± 0.05  *(0.0412)* | 0.23 ± 0.03  *(*0.3278)* | 0.77 ± 0.04  *(0.00008)* | 0.25 ± 0.02  *(*^$^*0.5848)* | 0.91 ± 0.08  *(0.0021)* |
| ***w*_10_ (10^-5^ nm^-1^)** | 177 ± 7 | 263 ± 7  *(0.0001)* | 195 ± 3  *(*0.0731)* | 205 ± 4  *(0.0232)* | 212 ± 5  *(*^$^*0.0067)* | 245 ± 10  *(0.0135)* |
| ***I*_ML1_** | 1 | 0.04 ±0.03  *(0.00004)* | 0.57 ± 0.04  *(*0.0013)* | 0.07 ± 0.04  *(0.0033)* | 1  *(*^$^*n/a)* | 0.05 ± 0.03  *(0.00007)* |
| ***I*_M3_** | 1 | 0.11 ± 0.03  *(0.00009)* | 0.78 ± 0.08  *(*0.0746)* | 0.39 ± 0.05  *(0.0404)* | 1  *(*^$^*n/a)* | 0.31 ± 0.03  *(0.0002)* |
| ***S*_M3_ (nm)** | 14.490 ± 0.003 | 14.489 ± 0.025  *(0.9636)* | 14.487 ± 0.006  *(*0.6307)* | 14.626 ± 0.010  *(0.0002)* | 14.488 ± 0.005  *(*^$^*0.6925)* | 14.609 ± 0.008  *(0.0006)* |
| ***w*_M3_ (10^-3^ nm^-1^)** | 5.3 ± 1.4 | 6.3 ± 2.1  *(0.7343)* | 9.6 ± 1.1  *(*0.0483)* | 11.4 ± 1.3  *(0.1089)* | 8.6 ± 1  *(*^$^*0.1063)* | 9.3 ± 1  *(0.0270)* |
| ***I*_M6_** | 1 | 0.99 ± 0.12  *(0.9548)* | 0.78 ± 0.06  *(*0.0406)* | 0.74 ± 0.15  *(0.7150)* | 1  *(*^$^*n/a)* | 0.87 ± 0.07  *(0.1804)* |
| ***S*_M6_ (nm)** | 7.240 ± 0.002 | 7.325 ± 0.024  *(0.0698)* | 7.246 ± 0.002  *(*0.0789)* | 7.328 ± 0.009  *(0.0026)* | 7.244 ± 0.005  *(*^$^*0.4898)* | 7.335 ± 0.005  *(0.0019)* |
| ***w*_M6_ (nm^-1^)** | 0.015 | 0.027 | 0.014 | 0.018 | 0.013 | 0.021 |
| ***S*_M1_ (nm)** | 43.6 | 42.1 | 43.1 | 41.7 | 43.2 | 41.9 |

**Table S2. X-ray parameters at 39°C and 7°C in intact quiescent and demembranated trabeculae in the absence and presence of Dextran.** Intensities are normalised by their values at 39°C in intact quiescent or demembranated trabeculae in the presence of Dextran, apart from *I*_11_ that is normalised by *I*_10_. *w*_10_, radial width of 1,0 reflection. *w*_M3_ and *w*_M6_ are radial widths for M3 and M6 reflection, respectively. Data are mean ± SE (apart from *w*_M6_ and *S*_M1_ where data are added) from n=5 or 4 intact trabeculae at 39°C and 7°C, respectively, and n=4 demembranated trabeculae. Values in brackets are P-values for paired or unpaired t-tests between 7°C and 39°C in each of the three groups of samples. * and ^$^ indicate P-values from unpaired t-tests between demembranated trabeculae in the absence and presence of 3% Dextran 38°C, respectively, with respect to intact trabeculae 39°C.
