# Supplementary material for: Cooling intact and demembranated trabeculae from rat heart releases myosin motors from their inhibited conformation
Source: J Gen Physiol. 2022 Jan 28;154(3):e202113029. doi: 10.1085/jgp.202113029 (PMC8823665; doi:10.1085/jgp.202113029)
Supplement: Table S3 — reports the transition temperatures of x-ray parameters. [file JGP_202113029_TableS3.docx]

|  | ***T*_0.5_ (°C)** | | |
| --- | --- | --- | --- |
|  | **Intact** | **Demembranated 3% Dex** | **Demembranated no Dex** |
| ***I*_M1_** | 19.9 ± 1.0* | 14.9 ± 0.5* | 23.8 ± 2.2 |
| ***I*_ML1_** | 19.7 ± 0.8* | 19.1 ± 0.6* | 23.7 ± 0.9 |
| ***I*_M3_** | 18.2 ± 0.7* | 19.3 ± 0.7 | 25.4 ± 1.6 |
| ***S*_M6_** | 12.7 ± 2.3^#^ | 14.7 ± 0.6^#^ | 18.2 ± 0.5 |

**Table S3. Transition temperatures of X-ray parameters.** The X-ray parameters were fitted with a Boltzmann curve y=*HT*+{(*LT*-*HT*)/[1+exp((*T*-*T*_0.5_)/d*T*)]}, where *LT* and *HT* are the values of the X-ray parameters at lowest and highest temperatures respectively, *T*_0.5_ is the half-maximal transition temperature and d*T* is the slope of the sigmoid. Intensities were normalized by the value at 39°C and 38°C in intact trabeculae and in demembranated trabeculae in the presence of Dextran, respectively (see Figs. S5D, 3C and 4D).

* indicates fittings where *LT* and *HT* were constrained to 0 and 1, respectively. ^#^ indicates fittings where *LT* was fixed to the value at the lowest temperature. Errors are obtained from the fitting procedure and do not indicate variability between trabeculae.
