# Supplementary material for: Cooling intact and demembranated trabeculae from rat heart releases myosin motors from their inhibited conformation
Source: J Gen Physiol. 2022 Jan 28;154(3):e202113029. doi: 10.1085/jgp.202113029 (PMC8823665; doi:10.1085/jgp.202113029)
Supplement: Table S4 — provides the model parameters for the best fit of the axial profile of the myosin (M) reflections. [file JGP_202113029_TableS4.docx]

|  |  | **Protocol** | ***T* (°C)** | ***N*** | ***hbz* (nm)** | ***n*_p_** | ***n*_d_** | ***d* (nm)** | ***ID* (nm)** | **y** |
| --- | --- | --- | --- | --- | --- | --- | --- | --- | --- | --- |
|  |  |  |  |  |  |  |  |  |  |  |
| **Intact trabeculae** | **M3H** | **Quiescent** | 39 | 4 | 80.0 ± 0.1 | 1 ± 0 | 48 ± 1 | 14.486 ± 0.005 | 845 ± 14 | 4.3 ± 2.0 |
|  |  |  | 28 | 5 | 80.2 ± 0.1  *(0.0353)* | 2 ± 1  *(0.0993)* | 46 ± 1  *(0.0069)* | 14.478 ± 0.004  *(0.0381)* | 818 ± 19  *(0.0455)* | 5.8 ± 2.4  *(0.3430)* |
|  |  |  | 22 | 5 | 80.3 ± 0.2  *(0.0225)* | 2 ± 1  *(0.0890)* | 47 ± 2  *(0.2411)* | 14.465 ± 0.014  *(0.0252)* | 840 ± 23  *(0.7529)* | 9.7 ± 5.0  *(0.0747)* |
|  |  |  | 18 | 5 | 80.2 ± 0.2  *(0.2215)* | 2 ± 1  *(0.2080)* | 39 ± 2  *(0.0001)* | 14.464 ± 0.011  *(0.0082)* | 722 ± 37  *(0.0008)* | 8.8 ± 4.2  *(0.0797)* |
|  |  |  | 7 | 4 | 86.2 ± 0.4  *(0.0001)* | 4 ± 3  *(0.1152)* | 29 ± 1  *(0.0001)* | 14.476 ± 0.005  *(0.0317)* | 618 ± 31  *(0.0002)* | 19.4 ± 3.7  *(0.0012)* |
|  |  | **Diastole** | 26.4 | # | 80.1 | 2 | 45 | 14.495 | 812 |  |
|  |  | **Peak force** | 26.4 | # | 86.1 | 6 | 30 | 14.515 | 666 |  |
|  | **M6H** | **Quiescent** | 39 | * | 81.2 | 1 | 85 | 7.24 | 770 |  |
|  | **M2H** |  | 39 | * | 80.2 | 1 | 26 | 21.54 | 699 |  |
|  | **M2L** |  | 39 | * | 86.4 | 1 | 16 | 22.943 | 517 |  |
|  |  |  |  |  |  |  |  |  |  |  |
| **Demembranated no Dextran** | **M3H** | **pCa 9** | 38 | 4 | 80.5 ± 0.1  *(^§^0.0057)* | 3 ± 1  *(^§^0.0663)* | 47 ± 2  *(^§^0.1328)* | 14.489 ± 0.012  *(^§^0.7561)* | 849 ± 19  *(^§^0.0261)* | 12.7 ± 3.7  *(^§^n/a)* |
|  |  |  | 26 | 4 | 80.5 ± 0.1  *(0.9088)* | 2 ± 1  *(0.1817)* | 41 ± 2  *(0.0086)* | 14.471 ± 0.006  *(0.0123)* | 751 ± 25  *(0.0086)* | 13.2 ± 3.0  *(0.6939)* |
|  |  |  | 22 | 3 | 80.4 ± 0.1  *(0.3662)* | 2 ± 2  *(0.4616)* | 35 ± 2  *(0.0003)* | 14.445 ± 0.019  *(0.0376)* | 671 ± 16  *(0.00005)* | 23.9 ± 6.3  *(0.0704)* |
|  |  |  | 18 | 4 | 80.5 ± 0.3  *(0.9085)* | 4 ± 1  *(0.2522)* | 41 ± 3  *(0.0493)* | 14.445 ± 0.011  *(0.0017)* | 779 ± 59  *(0.1323)* | 25.0 ± 3.2  *(0.0138)* |
|  |  |  | 9 | 4 | 90.6 ± 0.5  *(0.00004)* | 1 ± 0  *(0.0663)* | 48 ± 2  *(0.4228)* | 14.630 ± 0.011  *(0.000002)* | 862 ± 29  *(0.1541)* | 34.3 ± 13.0  *(0.0271)* |
|  |  |  |  |  |  |  |  |  |  |  |
| **Demembranated 3% Dextran** | **M3H** | **pCa9** | 38 | 4 | 80.5 ± 0.1  *(^§^0.0031)* | 4 ± 1  *(^§^0.0032)* | 49 ± 1  *(^§^0.4950)* | 14.486 ± 0.010  *(^§^0.9097)* | 889 ± 7  *(^§^0.0125)* | 9.5 ± 3.3  *(^§^n/a)* |
|  |  |  | 26 | 4 | 80.5 ± 0.1  *(0.1913)* | 2 ± 1  *(0.0060)* | 48 ± 1  *(0.4444)* | 14.480 ± 0.010  *(0.0743)* | 853 ± 22  *(0.0811)* | 10.9 ± 4.5  *(0.1083)* |
|  |  |  | 22 | 4 | 80.5 ± 0.3  *(0.6683)* | 2 ± 1  *(0.0060)* | 45 ± 2  *(0.0342)* | 14.476 ± 0.004  *(0.2114)* | 805 ± 34  *(0.0144)* | 12.2 ± 4.1  *(0.0227)* |
|  |  |  | 18 | 4 | 80.4 ± 0.2  *(0.3189)* | 2 ± 1  *(0.0138)* | 40 ± 1  *(0.0007)* | 14.460 ± 0.015  *(0.0049)* | 732 ± 19  *(0.0002)* | 13.4 ± 4.9  *(0.0192)* |
|  |  |  | 9 | 4 | 90.7 ± 0.4  *(0.00002)* | 2 ± 2  *(0.1616)* | 47 ± 2  *(0.2753)* | 14.638 ± 0.007  *(0.000007)* | 866 ± 28  *(0.1734)* | 31.8 ± 10.9  *(0.0127)* |
|  |  | **pCa 4.7** | 27 | $ | 90.9 | 1 | 49 | 14.653 | 885 |  |

**Table S4. Model parameters for the best fit of the axial profile of the myosin (M) reflections.**  Temperature, *T*; number of trabeculae, *N*; half-bare zone, *hbz*; proximal and distal layers indicating length of the array of contributing diffractors, *n*_p_ and *n*_d_, respectively; axial periodicity between adjacent layers of myosin motors, *d*; interference distance, *ID*; intensity scaling factor, *y*. Model run on data added from *N* = 6 (#), 4 (*) or 2 ($) trabeculae. Data in diastole, peak force and pCa 4.7 are from Brunello et al. (2020). Values in brackets are P-values for paired or unpaired t-tests between 39°C and the other four temperatures in each of the three groups of samples. *^§^* indicates P-values from paired t-tests between demembranated trabeculae in the absence or presence of 3% Dextran at 38°C and intact trabeculae at 39°C.

Brunello, E., L. Fusi, A. Ghisleni, S.J. Park-Holohan, J.G. Ovejero, T. Narayanan, and M. Irving. 2020. Myosin filament-based regulation of the dynamics of contraction in heart muscle. Proc. Natl. Acad. Sci. USA. 117:8177–8186. 10.1073/pnas.1920632117
